# Soybean genomics research community strategic plan: A vision for 2024–2028

**DOI:** 10.1002/tpg2.20516

**Published:** 2024-11-21

**Authors:** Robert M. Stupar, Anna M. Locke, Doug K. Allen, Minviluz G. Stacey, Jianxin Ma, Jackie Weiss, Rex T. Nelson, Matthew E. Hudson, Trupti Joshi, Zenglu Li, Qijian Song, Joseph R. Jedlicka, Gustavo C. MacIntosh, David Grant, Wayne A. Parrott, Tom E. Clemente, Gary Stacey, Yong‐qiang Charles An, Jose Aponte‐Rivera, Madan K. Bhattacharyya, Ivan Baxter, Kristin D. Bilyeu, Jacqueline D. Campbell, Steven B. Cannon, Steven J. Clough, Shaun J. Curtin, Brian W. Diers, Anne E. Dorrance, Jason D. Gillman, George L. Graef, C. Nathan Hancock, Karen A. Hudson, David L. Hyten, Aardra Kachroo, Jenny Koebernick, Marc Libault, Aaron J. Lorenz, Adam L. Mahan, Jon M. Massman, Michaela McGinn, Khalid Meksem, Jack K. Okamuro, Kerry F. Pedley, Katy Martin Rainey, Andrew M. Scaboo, Jeremy Schmutz, Bao‐Hua Song, Adam D. Steinbrenner, Benjamin B. Stewart‐Brown, Katalin Toth, Dechun Wang, Lisa Weaver, Bo Zhang, Michelle A. Graham, Jamie A. O'Rourke

**Affiliations:** ^1^ Department of Agronomy and Plant Genetics University of Minnesota St. Paul Minnesota USA; ^2^ USDA‐ARS Soybean & Nitrogen Fixation Research Unit Raleigh North Carolina USA; ^3^ USDA‐ARS Donald Danforth Plant Science Center St. Louis Missouri USA; ^4^ Division of Plant Science and Technology University of Missouri Columbia Missouri USA; ^5^ Department of Agronomy Purdue University West Lafayette Indiana USA; ^6^ Smithbucklin for the United Soybean Board St. Louis Missouri USA; ^7^ USDA‐ARS Corn Insects and Crop Genetics Research Unit Ames Iowa USA; ^8^ Department of Crop Sciences University of Illinois Urbana Illinois USA; ^9^ MU Institute for Data Science and Informatics University of Missouri–Columbia Columbia Missouri USA; ^10^ Department of Crop and Soil Sciences, and Institute of Plant Breeding, Genetics and Genomics University of Georgia Athens Georgia USA; ^11^ USDA‐ARS Soybean Genomics and Improvement Laboratory, Beltsville Agricultural Research Center Beltsville Maryland USA; ^12^ Farmer's Business Network St. Louis Missouri USA; ^13^ Roy J. Carver Department of Biochemistry, Biophysics and Molecular Biology Iowa State University Ames Iowa USA; ^14^ Department of Agronomy Iowa State University Ames Iowa USA; ^15^ Center for Applied Genetic Technologies University of Georgia Athens Georgia USA; ^16^ Department of Agronomy & Horticulture University of Nebraska Lincoln Nebraska USA; ^17^ Syngenta Slater Iowa USA; ^18^ Donald Danforth Plant Science Center St. Louis Missouri USA; ^19^ USDA‐ARS Plant Genetics Research Unit Columbia Missouri USA; ^20^ USDA‐ARS Soybean/Maize Germplasm, Pathology and Genetics Research Unit Urbana Illinois USA; ^21^ USDA‐ARS Plant Science Research Unit St. Paul Minnesota USA; ^22^ Department of Plant Pathology The Ohio State University Wooster Ohio USA; ^23^ Department of Biological, Environmental, and Earth Sciences University of South Carolina Aiken Aiken South Carolina USA; ^24^ USDA‐ARS Crop Production and Pest Control Research Unit West Lafayette Indiana USA; ^25^ Department of Plant Pathology University of Kentucky Lexington Kentucky USA; ^26^ Department of Crop, Soil and Environmental Sciences Auburn University Auburn Alabama USA; ^27^ Corteva Agriscience Johnston Iowa USA; ^28^ Department of Plant, Soil, and Agricultural Systems Southern Illinois University Carbondale Illinois USA; ^29^ USDA‐ARS Crop Production and Protection Beltsville Maryland USA; ^30^ USDA‐ARS Foreign Disease‐Weed Science Research Unit Ft. Detrick Maryland USA; ^31^ DOE Joint Genome Institute Lawrence Berkeley National Laboratory Berkeley California USA; ^32^ HudsonAlpha Institute of Biotechnology Huntsville Alabama USA; ^33^ Department of Biological Sciences University of North Carolina at Charlotte Charlotte North Carolina USA; ^34^ Department of Biology University of Washington Seattle Washington USA; ^35^ Bayer Crop Science St. Louis Missouri USA; ^36^ Inari Agriculture NV Zwijnaarde, Ghent Belgium; ^37^ Department of Plant, Soil and Microbial Sciences Michigan State University East Lansing Michigan USA; ^38^ School of Plant and Environmental Sciences Virginia Polytechnic Institute and State University Blacksburg Virginia USA

## Abstract

This strategic plan summarizes the major accomplishments achieved in the last quinquennial by the soybean [*Glycine max* (L.) Merr.] genetics and genomics research community and outlines key priorities for the next 5 years (2024–2028). This work is the result of deliberations among over 50 soybean researchers during a 2‐day workshop in St Louis, MO, USA, at the end of 2022. The plan is divided into seven traditional areas/disciplines: Breeding, Biotic Interactions, Physiology and Abiotic Stress, Functional Genomics, Biotechnology, Genomic Resources and Datasets, and Computational Resources. One additional section was added, Training the Next Generation of Soybean Researchers, when it was identified as a pressing issue during the workshop. This installment of the soybean genomics strategic plan provides a snapshot of recent progress while looking at future goals that will improve resources and enable innovation among the community of basic and applied soybean researchers. We hope that this work will inform our community and increase support for soybean research.

## Core Ideas

- Public and private investment is critical to advance and apply soybean genomics and related fields of research.
- Research priorities coevolve with the emergence of new opportunities and technologies available to researchers.
- The strategic plan was designed by and for soybean researchers working within various scientific sub‐disciplines.
- The plan summarizes the current state of knowledge and defines funding priorities for 2024–2028.

**Abbreviations**
AI: artificial intelligence
AMT: *Agrobacterium*‐mediated transformation
ASR: Asian soybean rust
BI/BreedBase: Breeding Insight/BreedBase
BPMV: *bean pod mottle virus*
CCMT: Cross‐Species and Comparative Multiomics Translation
CGH: comparative genomic hybridization
CNV: copy number variation
CO_2_
: carbon dioxide
CPSMV: cowpea severe mosaic virus
CRISPR: Clustered Regularly Interspaced Short Palindromic Repeats
DRTs: domestication‐related traits
EMS: ethyl methanesulfonate
FN: fast neutron
GenVarX: Genomic Variations Explorer
GO: Gene Ontology
GWAS: genome‐wide association study
ICS: isochorismate synthase
IDC: iron deficiency chlorosis
JGI: Joint Genome Institute
KEGG: Kyoto Encyclopedia of Genes and Genomes
LD: linkage disequilibrium
MADis: Multiple Alleles discovery
MG: maturity group
MU: University of Missouri
NLR: nucleotide‐binding leucine‐rich repeat
NMU: *N*‐methyl nitrosourea
NPGS‐GRIN: National Plant Germplasm System‐Germplasm Resources Information Network
NUST: Northern Uniform Soybean Tests
PI: plant introduction
PPI: protein–protein interaction
QTL: quantitative trait locus
RCR: red crown rot
SA: salicylate
SCN: soybean cyst nematode
scRNA: single‐cell RNA
SDS: sudden death syndrome
SIU: Southern Illinois University
SMV: *soybean mosaic virus*
SNP: single‐nucleotide polymorphism
SoyBase: SoybeanBase
SoyGEC: Soybean Genomics Executive Committee
SoyKB: Soybean Knowledge Base
STEM: Science, Technology, Engineering, and Mathematics
SUST: Southern Uniform Soybean Test
SWEET: Sugars Will Eventually bE Transported
TFs: transcription factors
TILLING: Targeting Induced Local Lesions in Genomes
TRD: taproot decline
UMN: University of Minnesota
USDA: United States Department of Agriculture
UST: Uniform Soybean Tests
VIGS: virus‐induced gene silencing
Wm82: Williams 82

## 1 INTRODUCTION

Prior to the advent of molecular biology, soybean [*Glycine max* (L.) Merr.] was considered a model plant for studies of plant physiology, contributing significantly to our basic knowledge of plant biosynthetic pathways, hormonal responses, and numerous other processes. Indeed, Sue Rhee (Carnegie Institute, California) indicated that much of the basic pathway information that was used initially to create the Arabidopsis Information Resource (TAIR; https://www.arabidopsis.org/) came from these basic studies done with soybean. However, during the molecular era, the model plant community focused on developing molecular tools for *Arabidopsis thaliana* and rice (*Oryza sativa* L.), while the soybean community instead focused on agronomic traits. The net effect is that soybean lagged in the development of modern molecular tools and their application. This greatly impacted efforts for soybean improvement, as the development of molecular resources and basic research enhances and enables downstream applications. Perhaps the best example of this is the completion of the soybean genome sequence, which had an initial basic research focus but now has widespread application for practical soybean improvement. Although slow to develop, it can now be claimed that the tools and approaches applicable to Arabidopsis research can be equally applied to the study of soybean.

While one can argue that it is appropriate for soybean researchers to focus on issues that impact farm productivity and profitability, such a narrow approach can have negative impacts. A clear case is funding from the National Science Foundation (NSF) Plant Genome Research Program, where conservative estimates suggest that, at least in the early years of this program, the majority of the funding went to support maize (*Zea mays* L.) research. This meant that maize growers did not have to invest their own funds into research and their lobbying efforts had a significant multiplying effect on maize improvement. During this time, only a handful of soybean NSF proposals were funded, though the number of such proposals submitted is unknown. There were no obvious lobbying efforts to try to capture public funding to support the priorities of the soybean community.

**FIGURE 1:**
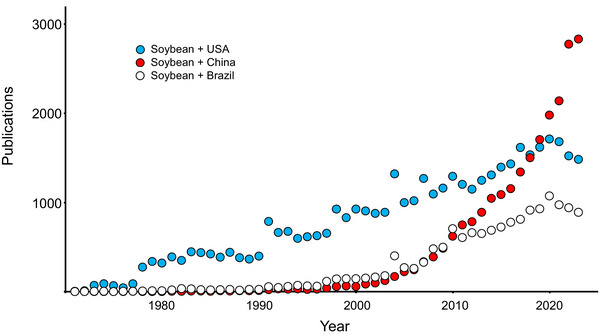
Number of publications by year estimated to be focused on soybean coming from researcher groups in different countries. The number of publications was identified from the ISI Web of Science database, using each respective search term as shown in the legend (“Soybean and USA,” etc.) (accessed March 26, 2024).

Today, we are facing another inflection point where the focus on soybean agronomic traits may be missing significant shifts that will negatively impact soybean growers. Specifically, there is a sharp rise in international competition within the soybean research community. As can be witnessed by several of the citations in this report and exemplified by Figure 1, China continues to invest heavily in soybean research. For example, a research hub in Wuhan, China contains at least five laboratories focused specifically on the soybean–rhizobium symbiosis, with many other labs around China conducting similar research. In comparison, there are probably only five laboratories in the entire United States conducting research on the soybean–rhizobial symbiosis. This is just one example; international laboratories are focused heavily on a wide variety of basic and applied questions using soybean as a crop model species. US‐based multinational corporations are already licensing technology from these international sources and are actively collaborating with these institutions. The net effect is that the United States is losing its preeminence in soybean research. US farmers will see the impact when they pay high premiums for seeds from these multinational corporations.

In addition to supporting research for practical soybean improvement, we also encourage a broadening of research efforts to increase overall funding, especially from public sources, to enlarge the soybean research community and highlight soybean as a model crop for plant research. The soybean research community needs the soybean farming community to lobby for increased public support of soybean research, especially in the face of growing international competition. An increase in funding, regardless of whether focused on discovery or application, will ultimately benefit soybean farmers by addressing critical questions related to soybean physiology, yield, development, and stress tolerance, as outlined in the reports below.

With this backdrop, the Soybean Genomics Executive Committee (SoyGEC) convenes a meeting for ∼50 US researchers working on soybean genetics, genomics, physiology, and related areas approximately every 5 years. The purpose of the meeting is to review recent accomplishments and discuss future priorities, culminating in a strategic plan for the community (e.g., Boerma et al., 2011; Jones & Stupar, 2017). The most recent meeting occurred in St. Louis, MO, USA, from November 30 to December 1, 2022. The report below outlines major accomplishments since the last strategic plan and defines a strategic plan for 2024–2028. The report is divided into subdisciplinary components: Breeding, Biotic Interactions, Physiology and Abiotic Stress, Functional Genomics, and Biotechnology. Bioinformaticians, including representatives from SoybeanBase (SoyBase) and Soybean Knowledge Base (SoyKB), were included within each of these teams and were later asked to draft sections on Genomic Resources and Datasets, and Computational Resources. While there are overlapping needs and interests among these groups, the report is presented within these thematic areas. Discussions at the meeting also focused on Training the Next Generation of Soybean Researchers, including efforts to bring more equity, diversity, and inclusion into the soybean research community. A summary of the team reports is presented below.

**Core Ideas**

- Public and private investment is critical to advance and apply soybean genomics and related fields of research.
- Research priorities coevolve with the emergence of new opportunities and technologies available to researchers.
- The strategic plan was designed by and for soybean researchers working within various scientific sub‐disciplines.
- The plan summarizes the current state of knowledge and defines funding priorities for 2024–2028.

## 2 BREEDING

Soybean is one of the most important crops in the world. With its high protein concentration, a complete amino acid profile, and a high proportion of unsaturated fatty acids, soybean is the world's largest source of animal feed protein and the second‐largest source of vegetable oil (Wilson, 2008). The average of on‐farm soybean yields in the United States is estimated to have increased by approximately 0.45 kg ha^−1^ per year from 1924 to 2022 (USDA‐NASS, 2022). However, soybean yield has been negatively affected by abiotic and biotic stresses, leading to significant yield losses in soybean production (Bradley et al., 2021). An additional challenge to continuous increases in seed yield is the negative correlation between seed yield and seed protein, which accounts for 60%–70% of the value of the soybean crop. Development of high‐yielding soybean cultivars with optimum seed compositions and resistance to abiotic and biotic stresses using innovative technologies is imperative to increasing the profitability and sustainability of the crop. The soybean breeding community has made remarkable accomplishments recently in developing new soybean cultivars and enhanced germplasm, discovering quantitative trait loci (QTLs)/genes for traits of importance, and utilizing new genomic and phenomic technologies to facilitate soybean breeding.

### 2.1 Breeding—Recent accomplishments

#### 2.1.1 Development of new germplasm and cultivars with novel genetics

In recent years, public soybean breeding programs in the United States have developed and released high‐yielding soybean germplasm/cultivars with improved seed composition, abiotic and biotic stress resistance, and herbicide tolerance. Specifically, released soybean cultivars and germplasm possess enhanced seed composition including high oleic and low linolenic acids, high protein and high oil contents, low oligosaccharides, high sucrose, and low seed phytate (Bhusal et al., 2022; Fallen et al., 2022; Florez‐Palacios et al., 2020; Lee et al., 2019; Li, Bachleda, et al., 2022; Li, Smith, et al., 2023; McNeece et al., 2020; Mian et al., 2023, 2021; Pantalone & Smallwood, 2018; Pantalone & Wyman, 2020; Pantalone et al., 2020, 2022; Smallwood et al., 2018). Based on special market needs, the community also developed soybean cultivars for specialty uses, including food grade, Natto, edamame, tofu, and black food type soybeans (Moseley et al., 2019; Singh, 2020a, 2020b, 2020c, 2020d; Zhang, Lord, et al., 2022).

Releases with resistance to biotic stresses including soybean cyst nematode (SCN), root‐knot nematode, frogeye leaf spot, stem canker, phytophthora root rot, aphids, white mold, and caterpillar resistance have also been made available (Diers et al., 2023; Fritz et al., 2023; Pantalone & Wyman, 2020; Pantalone et al., 2020, 2018, 2022; Ravelombola et al., 2023).

Some released soybean cultivars and germplasm have enhanced tolerance to abiotic stresses, including drought, heat, flooding, and iron deficiency chlorosis (IDC) (Fallen et al., 2023; Li, Smith, et al., 2023; Manjarrez‐Sandoval et al., 2020; Smallwood et al., 2024; Wu et al., 2024), and to herbicides such as glyphosate and LibertyLink (Li et al., 2021; Miranda., 2021b).

Many of these improved soybean cultivars have been commercialized by seed companies. Developed soybean germplasm has been shared with the public and private sectors to be used as breeding stocks. Since most cultivars released by public breeding programs are licensed directly to commercial seed companies without a publication or a Plant Variety Protection application, research publications currently fail to capture the impact of these releases.

#### 2.1.2 Mining soybean germplasm for genetic diversity and genetic improvement of yield and seed composition

Genetic diversity is important for the genetic improvement of soybean yield, seed composition, and other important traits. The North American soybean germplasm has a narrow genetic base where ∼75% of North American cultivars released from 1947 to 1988 were derived from 17 soybean ancestors and ∼50% were derived from only six ancestral lines (Gizlice et al., 1994). Research by Hyten et al. (2006) indicated that roughly 79% of rare alleles were lost during the introduction of soybeans to the United States. Mining exotic germplasm for beneficial alleles to develop enhanced soybean germplasm with exotic pedigrees can help soybean breeders exploit these alleles in their breeding programs to generate enhanced soybean germplasm. In recent years, public soybean breeders have developed high‐yielding, high‐protein germplasm and varieties with unique diversity from the germplasm collection (Bagherzadi et al., 2020, 2022; Eickholt et al., 2019; Florez‐Palacios et al., 2021; Gillen et al., 2019; Li, Bachleda, et al., 2022; Manjarrez‐Sandoval et al., 2018; Mian et al., 2021; Miranda, 2021a; Smith et al., 2020, 2019; Stewart‐Brown et al., 2018), where more than 70 additional accessions from the germplasm collection were identified and utilized in breeding programs. These releases impact soybean production in the United States and lay a solid foundation for future genetic improvement of soybean.

#### 2.1.3 Discovery and utilization of genomic tools to improve breeding efficiency

Using genome‐wide association or biparental mapping approaches with single‐nucleotide polymorphism (SNP) datasets, such as the SoySNP50K (Song et al., 2013), a number of QTLs/genes for important traits have been mapped or cloned. These include genes that control elevated seed protein concentration on chromosome 20 (Fliege et al., 2022; Goettel et al., 2022), *Phytophthora sojae* resistance genes *Rps11* (Wang, Chen, et al., 2021), *Rps12* (Sahoo et al., 2017), *Rps13* (Sahoo et al., 2021), and *Rps14* (Chen et al., 2021), SCN resistance gene *Rhg2* (Basnet et al., 2022), Southern stem canker resistance gene *Rdm3* (Menke et al., 2023), Frogeye leaf spot resistance genes *Rcs2* and *Rcs3* (McDonald, Buck, & Li, 2023a, 2023b), soybean rust resistance gene *Rpp7* (Childs et al., 2018), and genes controlling flowering time, maturity (Lu et al., 2020), seed coat luster (Zhang et al., 2018), and pod shatter (Zhang & Singh, 2020).

Datasets from SNP platforms ranging from 1K, 3K, 6K (Song et al., 2020), and 50K (Song et al., 2013, 2015) were developed and applied to breeding programs to characterize the soybean germplasm and for genomic selection and cross prediction (Table 1). The 50K assay was used to genotype the entire United States Department of Agriculture (USDA) Soybean Germplasm Collection containing approximately 20,000 accessions (Song et al., 2015) and delineate soybean genome‐wide haplotype blocks. The 50K SNP information was used to develop a smaller 6K platform (Song et al., 2020), which has proven useful for locating QTLs/genes controlling traits in biparental populations. Despite the success of the 50K and 6K assays, 3K and 1K platforms (Song et al., 2024) were developed to meet breeders’ needs for reduced marker density and costs. These platforms include markers associated with different traits to enhance early breeding selection of desirable progeny or genomic prediction. The 50K, 6K, 3K, and 1K assays form a series of nested SNPs, such that the 6K platform is a subset of the 50K platform, and so on. This nested design allows users to compare or merge existing datasets, or to impute datasets using common markers (Song et al., 2024). These SNP assays have been used by more than 50 laboratories around the world in both public and private sectors. The community also developed other assays for genotyping (Wang, Campbell, et al., 2023). Using SNP marker sets and materials from advanced yield trials or Uniform trials, models for genomic selection and cross‐prediction have been built and successfully deployed in soybean breeding programs (Miller, Song, & Li, 2023; Miller, Song, Fallen, et al., 2023; Stewart‐Brown et al., 2019). (Results of the Uniform trials can be accessed here: https://www.soybase.org/uniform_trials.php.) The marker sets have also been used to characterize soybean germplasm (Stewart‐Brown et al., 2020) and perform genome‐wide association studies (GWAS) for traits of importance (Chamarthi et al., 2021; McDonald, Buck, Song, et al., 2023; Shook et al., 2021; Steketee et al., 2020; Walker et al., 2022; Zimmer et al., 2021).

**TABLE 1:** Single‐nucleotide polymorphism (SNP) platforms developed to enable soybean breeding and genetics.

Platform	Utility and/or function	Manufacturer	Reference
SoySNP50K	Genotyped 20,000 accessions	Illumina Inc.	Song et al. (2015)
BARCSoySNP6KSoySNP6K	Locate QTLs/genes in biparental populations	Illumina Inc.	Song et al. (2020)
SoySNP3K	Reduced marker density and cost for breeding applications, marker imputation, and inclusion of trait‐associated markers	Illumina Inc.	Song et al. (2024)
SoySNP1K	Reduced marker density and cost for genomic selection and prediction, marker imputation, and inclusion of trait‐associated markers	Agriplex Genomics Inc.	Song et al. (2024)

Based on the mapped QTLs or cloned genes for traits of importance, the community has successfully deployed marker‐assisted selection in soybean breeding programs. This includes selecting for traits such as reduced Kunitz trypsin inhibitor (Rosso et al., 2021), charcoal rot resistance (Pawale et al., 2019), *soybean mosaic virus* (SMV) resistance (Karthikeyan et al., 2023), carbohydrate profiles (Hagely et al., 2020), resistance to phytophthora rot and powdery mildew (Ramalingam et al., 2020), hard‐seededness and pod shattering (Kumawat et al., 2021), protein content, and oleic and linolenic acids (Darr et al., 2020; McDonald, Bilyeu, et al., 2023).

#### 2.1.4 Development and utilization of image‐based phenotyping technologies in field, greenhouse, and laboratory

In recent years, the community has developed image‐based phenotyping technologies and deployed them in fields, greenhouses, and laboratories for traits of importance to support gene discovery and soybean breeding. The traits consist of canopy coverage (Moreira et al., 2019), sudden death syndrome (SDS) (Rairdin et al., 2022), resistance to soybean frogeye leaf spot (McDonald et al., 2022), soybean root phenotyping (Falk et al., 2020), soybean maturity (Trevisan et al., 2020), drought tolerance (Kim et al., 2022), IDC (Dobbels & Lorenz, 2019), nodulation (Carley et al., 2023), and soybean growth (Zhu, Sun, et al., 2020).

#### 2.1.5 Impact of public breeding efforts

Improved soybean cultivars have been commercialized by private or public entities, which benefit the soybean growers. Enhanced soybean germplasm lines developed in public breeding programs have been shared across public and private breeding programs for soybean research and cultivar development. Innovations and methodologies generated from public soybean breeding programs have helped improve breeding efficiency and accelerate genetic gains. Undergraduate and graduate students as well as postdocs trained in soybean breeding programs have become part of a skilled professional workforce in both academia and industry.

### 2.2 Breeding—Priorities (2024–2028)

#### 2.2.1 Germplasm/cultivar development

Development of new and improved soybean germplasm and cultivars is necessary to sustain soybean production in the United States. In order to continuously increase the rate of genetic gain and improve seed composition and resistance to abiotic and biotic stresses, soybean breeding emphasizes the following priorities: effective use of genetic diversity, incorporation of novel or improved traits into new germplasm and cultivars, optimizing seed composition while increasing yield, development of climate‐resilient germplasm, and development of cultivars for commodity soybean production as well as broader food uses such as meat substitutes.

#### 2.2.2 Utilization of genomic tools to improve breeding efficiency

Molecular breeding has become a powerful tool in supporting soybean breeding, and continued emphasis of this tool will greatly improve breeding efficiency. The following are priorities for molecular breeding: new QTLs and genes for economically important traits; gene‐specific or diagnostic DNA markers for effective marker‐assisted selection; high‐throughput, low‐cost genotyping technology and platforms; a national database to provide marker–trait association for genomic prediction; and optimization and deployment of genomic prediction/selection in soybean breeding programs.

#### 2.2.3 Development and deployment of innovative breeding technologies

Innovative breeding technologies are desired, including the following: direct use of genome editing technology in soybean breeding programs; new methodologies to effectively produce soybean hybrids for production; deployment of high‐throughput/precision phenotyping methods for traits of importance, including developmental and seed composition traits; deployment of comprehensive predictive models including genotypic, phenotypic, and environmental data for important traits; and advances in understanding the impact of the microbiome on soybean yield.

#### 2.2.4 Resources and sustainability

To sustain plant breeding efforts in public breeding programs, the following priorities are needed: improving the infrastructure of land‐grant universities and upgrading breeding equipment; continuing to build public and private partnerships and strengthen collaboration; and establishing a high‐throughput genotyping center to support public breeding efforts. Germplasm produced in the public sector is frequently shared with private seed companies via material transfer agreements. However, the impacts of such shared resources are often poorly documented. A method for public documentation of the use and impacts of public germplasm needs to be developed. The scope of training new plant breeding professionals needs to increase due to an acute shortage of qualified candidates to fill current and future vacancies.

## 3 BIOTIC INTERACTIONS

One overarching goal of the previous strategic plan was to improve and ensure accessibility to soybean genomic resources to facilitate research progress (Jones & Stupar, 2017). The implementation of strategies prioritized in the 2017 report had a significant impact on different areas of soybean biology, particularly in the generation of genomics data and tools to advance the fight against soybean pests and diseases.

### 3.1 Biotic interactions—Recent accomplishments

Significant efforts were dedicated to identifying new sources of resistance, resulting in a wealth of new genetic resources. A recent survey accounted for more than 800 resistance genes/loci and major QTLs for 28 of the most important soybean diseases caused by nematodes, oomycetes, fungi, bacteria, and viruses (Lin et al., 2022). Similarly, at least 49 SNPs associated with soybean aphid resistance were identified in 69 plant accessions (plant introduction [PI]) through GWASs (Natukunda & MacIntosh, 2020). By providing highly linked markers for progeny selection, these QTLs and associated SNPs will allow breakage of tight linkage between desired resistance traits and adjacent undesirable alleles. Improvements in the assembly and annotation of the *G. max* ‘Williams 82’ (Wm82) reference genome (Espina et al., 2024; Garg et al., 2023; Valliyodan et al., 2019; Wang, Zhang, et al., 2023), the completion of other fully annotated *G. max* genomes (e.g., Kim, Lee, et al., 2021; Shen et al., 2019), and access to genetic material and genomic resources in wild soybean (Patil et al., 2019; Valliyodan et al., 2019; Xie et al., 2019) and other closely related *Glycine* species (Liu, Chang, et al., 2018) have also increased the repertoire of available resistance genes that can be incorporated into breeding and biotechnology approaches to manage pests and diseases. These novel sources of resistance will become essential to produce the next generation of resistant soybean germplasm.

These resources also allowed the dissection of soybean defense mechanisms and highlighted the presence of novel defense strategies that have not been previously described in other plant species. For example, none of the known loci associated with resistance to SCNs contains the classical nucleotide‐binding leucine‐rich repeat (NLR) genes typically associated with disease resistance loci. Cloning of characterization of *rhg1‐b*, the most widely used SCN resistance locus, identified genomic and mechanistic surprises (Bent, 2022). Three genes are associated with resistance in this locus: a transporter (AAT_Rhg1_), a protein involved in membrane fusion (α‐SNAP_Rhg1_), and a wound‐inducible protein of unknown function (WI12). Interestingly, amplification of this multigene block is associated with resistance since most susceptible soybean germplasm carries one copy of the three‐gene *Rhg1* block, while protection against SCN is correlated with higher copy number in resistant accessions (Bent, 2022; Patil et al., 2019). Functional analyses indicate that the products of the three genes participate in different processes that lead to resistance, suggesting that “*Rhg1* is truly a multimechanism SCN resistance gene stack” (Bent, 2022). The identification of *GmSNAP11* as the candidate gene responsible for resistance at the *Rhg2* locus (Basnet et al., 2022; Shaibu et al., 2022) and that loss of function mutations in *GmSNAP02* also confers resistance to SCN (Usovsky et al., 2023) indicate that alterations in vesicular transport are important components of the plant defense mechanism against this nematode.

Another major SCN resistance gene, Rhg4 or GmSHMT08 (Glyma.08g108900), encodes a serine hydroxymethyltransferase responsible for serine and glycine interconversion, suggesting another unique mechanism of resistance (Liu et al., 2012). Whole‐genome re‐sequencing of a diverse panel of soybean accessions showed that, as is the case for *Rhg1*, resistance to SCN is correlated with a higher copy number of *Rhg4* loci (Patil et al., 2019).

Most of the available commercial varieties carry *Rhg1* (primarily) or *Rhg4*, and different sources of resistance are needed for the sustainable management of SCN. Genetic characterization of the locus conferring SCN resistance in PI567516C, an exotic soybean accession, identified several candidate genes, and remarkably none show similarity to the proteins associated with Rhg1 or Rhg4, suggesting a novel mechanism of resistance that provides protection against multiple SCN races (Zhou, Song, et al., 2021). Other work has laid out the native SCN resistance loci currently available in North American public soybean breeding programs, including some that were previously unknown (Mahmood et al., 2023).

Genome evolution and rearrangements of genomic sectors have also led to other innovations. The *Rps11* locus confers resistance to *P. sojae*, and it was recently shown that this locus encodes an unusually large NLR gene with broad‐spectrum resistance to at least a dozen *P. sojae* races (Wang, Chen, et al., 2021). *Rps11* is included in a genomic region containing a cluster of large NLRs of single origin in soybean with phylogenetic diversification among soybean varieties. These novel NLRs are likely to contribute to novel mechanisms that provide a broad‐spectrum resistance to other pathogens. In addition to NLRs, novelty in cell surface receptors has been described in soybean. For example, polymorphisms in flagellin receptors acquired after gene duplications also allowed soybean to recognize *Ralstonia solanacearum*, a wilt disease‐causing bacterium that can infect a wide range of plants, and functional and mechanistic analyses indicate that this resistance can be transferred from soybean to other crops (Wei et al., 2020).

Gene pyramiding has been a common tool utilized in plant breeding for decades, and this practice has also been proposed as a way to increase the durability of resistance (R) traits (Mundt, 2018). In soybean, gene pyramids have been used to increase resistance to several pathogens, including SDS, SMV, SCN, and Asian soybean rust (ASR) (reviewed by Lin et al., 2022), and to overcome virulent soybean aphid biotypes (e.g., Diers et al., 2023; Wiarda et al., 2012). While the effects of pyramiding *R* genes at the phenotypic level are clear, the molecular mechanisms underlying the increase in resistance are often poorly understood. Recently, Natukunda et al. (2021) demonstrated that the increased resistance against soybean aphids in a *Rag1*/*Rag2* pyramid is likely due to a synergistic effect manifested at the transcriptional level. Pyramid plants mount a unique response not triggered in plants carrying either *Rag* gene individually, and it was proposed that this outcome is the result of specific combinations of transcription factors (TFs) that occur only in the pyramid. This unique response exposes aphids to different selective pressures than plants with only *Rag1* or *Rag2*, likely increasing the durability of these *R* traits.

Fully sequenced reference genomes, and in some cases even pangenomes, are available for many of the main soybean pathogens and pests, including SCN (Masonbrink, Maier, Muppirala, et al., 2019, Masonbrink, Maier, Seetharam, et al., 2019), soybean aphid (Giordano et al., 2020; Mathers, 2020; Wenger et al., 2020), *Phakopsora pachyrhizi* (Gupta et al., 2023), *P. sojae* (Zhang, Liu, et al., 2019), *Pythium* spp. (Fernandes et al., 2019; Lévesque et al., 2010), and *Fusarium virguliforme* (Srivastava et al., 2014). These resources have facilitated the identification of pathogenicity factors and effectors and guided population and genetic diversity analyses. These advances have contributed to our understanding of the pathosystems and can aid in the identification of new targets for disease management. New phenotyping tools, better diagnostics, and defined geographic ranges for major soybean diseases have also contributed to basic knowledge and management practices (Hale et al., 2023; Mena et al., 2022; Roth et al., 2020).

Biotechnological advances achieved in the last few years promise to improve management practices. For example, a recent screen of nematicidal activities from soil bacteria identified a *Bacillus thuringiensis* delta‐endotoxin, Cry14Ab, that can control SCN in transgenic soybean (Kahn et al., 2021). This approach can be particularly impactful, given the prevalence of SCN and the current overreliance on PI88788‐derived resistance for control, and the trait was recently released in commercial varieties. A strategy with a much broader application has the potential to revolutionize pest management. Kourelis et al. (2023) produced hybrid receptors that combined rice‐derived Pik NLRs that normally recognized fungal effectors with camelid antibody fragments (nanobodies) that recognized green fluorescent protein or mCherry fluorescent protein. The resulting hybrid proteins (“pikobodies”) were expressed in planta and were able to trigger an immune response when the plants were challenged with the corresponding fluorescent protein. Since antibodies (and thus nanobodies) can be raised against almost any antigen, it should be possible to produce pikobodies that recognize any pathogen or pest that produces effectors that are translocated inside plant cells. This approach has not yet been tested in soybean, but it should be a priority, particularly for researchers working on emerging pathogens for which host plant resistance has not yet been identified.

While this section primarily addresses diseases and pests, it is important to consider also other biotic interactions that are beneficial to soybean. Rhizobia nodulation and its role in nitrogen fixation have been subjects of study for decades (reviewed in Du et al., 2023). Other plant–microbe interactions that are also fundamental to efficient plant performance are now receiving deserved attention. The nodule bacteriome contains non‐rhizobia bacteria, and its composition is dependent on soybean cultivar and changes in parallel with amino acid content in response to environmental stress, suggesting potential roles for those atypical bacteria (Sharaf et al., 2019). Microbes also have an important role outside of nodules. Analyses of the soybean rhizosphere microbiome indicated that the microbial community associated with the plant was dominated by Proteobacteria, Acidobacteria, Actinobacteria, and Bacteroidetes, but soil type and plant genotype had a strong influence on the microbes recruited from the soil to the rhizosphere (Liu et al., 2019). Prediction of the metabolic capacity of the root‐associated microbes suggested that pathways associated with xenobiotic degradation, plant–microbe interactions, and nutrient transport are enriched (Liu et al., 2019). Perhaps expected, the soybean rhizosphere microbiome is also affected by farming practices (Agyekum et al., 2023). Importantly, the rhizosphere microbiome can shape rhizobia–plant interactions and affect the composition of the nodule bacteriome (Han et al., 2020). Although the effect of rhizobia on yield is well understood, correlations between microbiome diversity or individual operational taxonomic units and yield are less evident (Niraula et al., 2022; Sharaf et al., 2019). While most studies have focused on the rhizosphere, much less is known about the microbiome associated with non‐root tissues, though studies have shown that spatial (organ/tissue) and temporal variables, as well as soybean genotype and environment, strongly influence the microbe species that can colonize each soybean niche (Moroenyane et al., 2021; Yang et al., 2023).

### 3.2 Biotic interactions—Priorities (2024–2028)

While we have generated a plethora of genetic and genomic resources, our mechanistic understanding of soybean defense pathways is lacking. We need to understand the molecular mechanisms that mediate the deployment of defenses against pests and pathogens if we are to develop predictive models that take full advantage of the genomic resources to connect the genome to the phenome.

Importantly, there are key differences in the soybean defense response with respect to other plants like Arabidopsis, tomato (*Solanum lycopersicum* L.), and tobacco (*Nicotiana tabacum* L.), traditionally used as models to study plant–pathogen interactions. The best characterized plant resistance genes are members of the NLR protein family. NLRs that recognize pathogen effectors are known as sensor NLR, and these receptors require interaction with other NLRs known as helper NLRs to initiate immune signaling (Bonardi et al., 2011). Recently, it was discovered that helper NLRs in their activated state form a structure known as “resistosome” that mediates defense signaling and hypersensitive responses (Bi et al., 2021; Jacob et al., 2021). However, some of the best studied helper NLRs are not conserved in soybean (Gong et al., 2022; Wu et al., 2017). Similarly, the antagonism between jasmonate and salicylate (SA) signaling has been well established in Arabidopsis (Pieterse et al., 2012); however, the interaction of these hormone signaling pathways does not seem to be antagonistic in soybean (Selig et al., 2016; Singh et al., 2011; Studham & MacIntosh, 2013). Interestingly, while the isochorismate synthase (ICS) pathway is the dominant contributor of defense‐related SA in Arabidopsis, both ICS and phenylalanine ammonia lyase are equally important in soybean (Shine et al., 2016). Given the large amount of genetic and genomic resources available for soybean, in particular, as related to resistance to pests and pathogens, and the identification of important differences in defense responses between soybean and model plant systems, we propose that efforts should be made to establish soybean as a true model organism for plant–pathogen research.

Most studies have generally focused on transcriptome analyses or early signaling events while studying soybean–pathogen or soybean–pest interactions. On the other hand, the biochemical and physiological changes that make the plant a less favorable host during the immune response have been understudied. For example, the biochemistry of isoflavones has not received much attention in the last few decades, despite their essential role in nodulation (Subramanian et al., 2006) and as antimicrobials and insect deterrents (Hohenstein et al., 2019; Lygin et al., 2010). Several studies have analyzed genetic diversity controlling total isoflavone content; however, the full complement of isoflavonoids present in the soybean metabolome and their potential functions have not been characterized. Other phytoalexins, such as terpenoids, saponins, or alkaloids, have received even less attention. Modification of physical barriers to infection and feeding, such as cuticle (and extracellular waxes) and cell wall, and plant mechanisms that modify the distribution of nutrients away from the pest or pathogen, can have a significant impact on plant productivity in addition to their important role in defense; thus, these areas of research should be developed.

Pests and pathogens continuously evolve mechanisms to escape host recognition and to suppress defenses; thus, single *R* genes have a “shelf life” once deployed, and development of appropriate management strategies is essential to extend the durability of resistance traits (Brown, 2015; Rimbaud et al., 2021). In addition, novel sources of resistance should still be identified, in particular taking advantage of the availability of genomic information from wild relatives. Although many sources of resistance have been identified in the last few years, few *R* genes have been cloned and characterized. These steps are crucial to accelerate biotechnological applications. Importantly, genes that increase susceptibility to pests and pathogens (Koseoglou et al., 2022) are understudied, and there should be a renewed effort to characterize them in soybean, as they could be ideal targets for gene editing technologies that could more easily overcome regulatory hurdles for utilization. Plant–pathogen/pest interactions are significantly influenced by abiotic stresses, as famously depicted by the “disease triangle” model (Velásquez et al., 2018). This aspect of plant disease is particularly important in view of the current global environmental changes caused by natural and human activities. Thus, a renewed focus on the combined effect of biotic and abiotic factors on soybean diseases is needed. These studies should also include the effects of these factors on the soybean‐associated microbiome.

Efforts to understand resistance and susceptibility traits and their underlying mechanisms will depend on the development of more effective and rapid tools for functional analysis or individual genes and gene families that currently limit our ability to pursue many high‐throughput functional analyses. Bioinformatics tools that leverage pangenome information to understand disease development and soybean defense mechanisms should also be improved and made widely available. These advances will benefit and should be favored by the establishment of improved infrastructure to access public datasets (genomes, effectomes, metabolomes, proteomes, etc.) and the tools to mine them. Repositories for genetic material, including soybean accessions and mutant collections, dataset integration, and pathogen repositories, are also essential and have been an unrealized priority for many years.

It is also important to focus on emerging pests and pathogens. For example, root‐knot nematodes (*Meloidogyne* spp.) have become an important pest of soybean with a significant impact on yield in places where they are endemic (Gorny et al., 2021). GWAS and QTL analyses have identified potential sources of resistance to these parasitic nematodes (Alekcevetch et al., 2021; Fallen et al., 2022; Li, Bachleda, et al., 2022; Pantalone & Wyman, 2020; Ravelombola et al., 2023), and plants overexpressing either the pathogenesis‐related protein GmPR10 or the expansin GmEXPA1 have increased resistance to root‐knot nematodes (Arraes et al., 2022; Basso et al., 2023). However, the mechanisms underlying soybean resistance to these species are still unknown.

Some other emerging soybean diseases in the United States worth mentioning are red crown rot (RCR) and taproot decline (TRD). RCR, which causes defoliation and early plant maturity, is reported to cause 25%−30% yield loss (Kleczewski et al., 2019). This root disease, caused by the soilborne fungus *Calonectria ilicicola*, results in rot and dark red discoloration on the stem portion closest to the soil (Crous et al., 1993). Red reproductive structures and white fungal growth can be observed on the lower stem and roots. Leaf damage, attributed to phytotoxin production by the fungus, is observed in the form of yellow discoloration and death of leaf tissue, while leaf veins remain green (Ochi et al., 2011). The disease is seed‐transmitted because it can spread via microsclerotia (Randall‐Schadel et al., 2001). Breeding for resistance has been a challenge because of the observed low levels of resistance among screened genotypes and the lack of comprehensive resistance screening of US cultivars (Jiang et al., 2020). Currently, there are no commercial soybean cultivars with resistance to RCR available in the United States. Histopathological studies show that the plant prevents fungal invasion through periderm formation and occlusion of xylem parenchyma cells (Yamamoto et al., 2017). While soybean producers in Louisiana and Mississippi have dealt with this disease for years, it appears to be moving northwards in recent years (Kleczewski et al., 2019; Neves et al., 2023).

TRD is caused by the fungus *Xylaria necrophora* and is also referred to as “dead man's fingers” because of the appearance of the fungal stromata (Allen et al., 2017). TRD causes wilting and interveinal chlorosis, followed by necrosis in foliar tissue, and this has been linked to phytotoxic secondary metabolites produced by the fungus in the roots (García‐Méndez et al., 2016). In addition, black fungal stroma are typically found embedded in the taproot and sometimes in the lateral roots (Allen et al., 2017). Symptoms are observed from early vegetative stage (V6) to full seed set (R6). Annual yield losses due to TRD have been reported to range from 0.2% to 1.5% (Allen et al., 2019). Greenhouse and field studies have detected TRD resistance in some commercially available soybean cultivars (Purvis, 2019). However, the patchy nature of the disease in fields can pose a challenge for trials. Currently, TRD has been reported in Alabama, Arkansas, Louisiana, Mississippi, Missouri, and Tennessee.

Finally, a better understanding of the economic value of disease‐resistance traits beyond yield improvement will necessitate the integration of multidisciplinary teams (including plant pathologists, breeders, economists, and social scientists among others) to increase support and drive advances in this area of research in the next quinquennial.

## 4 PHYSIOLOGY AND ABIOTIC STRESS

### 4.1 Physiology and abiotic stress—Recent accomplishments

Recent abiotic stress work has leveraged decades of physiological research on drought response mechanisms and tolerance traits alongside quantitative genetics to pinpoint loci that are associated with soybean drought responses. The visually accessible phenotype that breeders have long used to screen for potential drought tolerance, slow leaf wilting in dry conditions, could potentially be replaced with aerial measurements of canopy temperature as an indicator of transpiration (Bai & Purcell, 2018). This phenotyping strategy is based on the finding that a moderate water use strategy may conserve soil moisture water and enable continued productivity during periods of low precipitation (King et al., 2009), and it offers an advantage over manual wilting ratings in being objective, quantitative, and high throughput. In terms of genetics, multiple QTLs controlling slow wilting have been identified in recent years (Menke et al., 2024; Ye et al., 2020). Ye et al. (2020) also identified a physiological mechanism of water conservation in the early maturity groups (MGs) and confirmed yield benefits of slow wilting traits in near‐isogenic backgrounds under rainfed conditions.

High water use efficiency is advantageous in dry conditions (Leakey et al., 2019), and physiologist‐led groups have used this knowledge to identify loci associated with this trait as well as high stomatal conductance estimated via stable isotope analysis (Bazzer et al., 2020; Kaler et al., 2018, 2017). A desirable combination of traits for drought tolerance would include a moderate to high baseline water use efficiency as well as a large capacity to increase water use efficiency under drier conditions. These traits were quantified among nearly 500 soybean accessions using regression analysis for carbon isotope ratio as an indicator of water use efficiency in a particular genotype–environment combination (Chamarthi et al., 2023). Soybean has a broad range of phenotypic plasticity for water use efficiency, and seven genetic loci were associated with plasticity, indicating potential for optimizing phenotypic plasticity via breeding or biotechnology.

Flood resilience is a major concern in soybean production, and recent work has elucidated some of its genetic and physiological underpinnings. Ye et al. (2018) and Wu et al. (2020) identified several genetic loci associated with better visual ratings after waterlogging or partial submergence, and Dhungana et al. (2020) mapped QTLs controlling chlorophyll and biomass responses to seedling submergence. A favorable allele on chromosome 3 improved root growth in both control and waterlogged conditions and may impact auxin‐regulated root development, an important mechanistic discovery (Ye et al., 2018). Efficient phenotyping for flood resilience remains a challenge, and new screening techniques, including a hydroponic assay and artificial intelligence (AI)‐assisted unmanned aerial vehicle image analysis, will enable more rapid genetic advances in the coming years (Harrison et al., 2022; Zhou, Mou, et al., 2021).

Field‐based research on belowground organs lags in comparison to aboveground studies due to the lack of technology for rapid and/or nondestructive belowground measurements. Nonetheless, Dhanapal et al. (2020) phenotyped soybean root crowns in nearly 300 genotypes in multiple field environments. This labor‐intensive study quantified broad phenotypic diversity in soybean root system architecture in the field and identified SNPs associated with root system architecture, which can play an important role in drought tolerance. Similar diversity and QTL analysis of root system architecture was also conducted in controlled greenhouse conditions (Prince et al., 2019), revealing a candidate gene for lateral root number. Furthermore, a two‐dimensional pouch growth system was recently used to phenotype root system architecture traits in controlled conditions (Chandnani et al., 2023), revealing additional candidate genes.

IDC presents another significant abiotic stress challenge for soybean production, particularly in the Upper Midwest (Hansen et al., 2003). IDC is characterized by symptoms such as interveinal chlorosis, stunted growth, and, in severe cases, necrosis. To manage IDC, agronomists use various strategies including seed treatments, foliar applications, soil iron chelates, and, most effectively, selecting resistant cultivars (reviewed in Merry et al., 2022). However, despite the preference for varietal resistance, no soybean varieties exhibit complete resistance to IDC. The genetic control of IDC resistance is complex, though several key QTLs have been identified (Merry et al., 2022). Noteworthy QTLs include one on chromosome 3 (Kohlhase et al., 2024; Lin et al., 1997; Peiffer et al., 2012), where the low‐resistance allele appears to be rare among elite breeding germplasm (Merry et al., 2019), and another on chromosome 5 (Assefa et al., 2020; Merry et al., 2019; O'Rourke et al., 2021). Confirming the causal polymorphisms associated with these QTLs could greatly enhance our understanding of the molecular and physiological mechanisms of IDC resistance. Gene expression and physiology studies suggest that the phenylpropanoid synthesis pathway, which regulates fluorescent root exudate compounds, might play a critical role (Waters et al., 2018), but numerous other mechanisms ranging from proton extrusion to improved iron transport to seed iron content all likely contribute to overall resistance (reviewed in Merry et al., 2022). Advances in image‐based phenotyping are improving the precision and sensitivity of IDC resistance measurements and capturing temporal changes (Bai et al., 2018; Dobbels & Lorenz, 2019; Naik et al., 2017). Given the spatial variability of IDC in the field, integrating advanced phenotyping with robust experimental design and analysis (Xu, Cannon, et al., 2022) will be crucial for accurately estimating genetic effects and advancing both mapping and physiological studies in the future.

The physiology community has begun to explore variations in heat stress responses among soybean genotypes and explore combinatorial effects of heat stress in the field. Diverging photosynthetic responses to temperature among genotypes have been observed in young plants in a greenhouse (Herritt & Fritschi, 2020) and in the field during reproductive development (Ortiz et al., 2022). Building on prior work that examined interactions between season‐long temperature elevation and future atmospheric carbon dioxide (CO_2_), an open‐air field study found that intense heat waves during reproductive development reduced soybean yield in three out of four trials, even at future CO_2_ (Thomey et al., 2019). Another open‐air field experiment found that season‐long elevated temperature reduced yield similarly in both elevated ozone and ozone‐scrubbed air (Burkey et al., 2020). While these studies are valuable advances in soybean abiotic stress research, the logistical difficulty of elevating temperature in the field has limited our understanding of soybean heat stress physiology at different developmental stages and among diverse genotypes.

Soybean's C3 photosynthesis is not optimized, with over 40% of potential photosynthetic efficiency lost to photorespiration and other biochemical inefficiencies in C3 plants (Walker et al., 2016; Zhu et al., 2010). Synthetic biology approaches to improve photosynthetic efficiency in soybean are in development (e.g., De Souza et al., 2022; Harvey et al., 2022). If such approaches successfully increase soybean's photosynthetic capacity, a concurrent increase in nutrient acquisition may be required to support greater productivity and yield.

Although soybean is often considered to meet its own nitrogen needs, biological nitrogen fixation has not fulfilled soybean's nitrogen requirement in high‐yielding conditions (Ciampitti & Salvagiotti, 2018). Furthermore, seven decades of soybean breeding did not increase the proportion of soybean nitrogen derived from fixation (Donahue et al., 2020), and higher reliance on nitrogen fixation was not predictive of yield (de Borja Reis et al., 2021). These findings highlight the need for improved biological nitrogen fixation combined with a greater understanding of source and sink controls on nitrogen metabolism for sustainable improvements in soybean productivity and quality. In controlled conditions, nitrogen fixation was increased by overexpression of a ureide transporter, *Phaseolus vulgaris* ureide permease 1 (*PvUPS1*) (Carter & Tegeder, 2016). Further work with these transformants demonstrated that ureide transport was not only stimulated in the nodule, but also in leaves, and that plants also increased photosynthesis and transport of photoassimilate in shoot tissues (Lu et al., 2022; Thu et al., 2020). While these experiments were limited to controlled environments, they highlighted the potential for alterations in whole‐plant nitrogen transport to stimulate nitrogen fixation and productivity.

Soybean seed quality as defined by end‐use preferences has declined over several decades of breeding for yield. The identification of loci associated with seed protein concentration, as well as alleles for high oleic acid content, has aided seed composition improvement through breeding (e.g., Pham et al., 2011; Prenger, Yates, et al., 2019). Other seed composition optimizations, however, will require a mechanistic understanding of seed metabolism throughout development and consideration of spatial organization. Kambhampati et al. (2021) recently demonstrated that understanding and manipulating the temporal regulation of seed metabolism may be key to optimizing difficult‐to‐breed seed composition traits and turnover of valuable storage products, such as lipid late in development can result in a less valuable seed (Aznar‐Moreno et al., 2022). Changes in the subcellular location of steps in metabolism can have equally important consequences for the composition of final storage reserves (Morley et al., 2023). Furthermore, although both protein and oil are known to be impacted by the environment (Assefa et al., 2019; Rotundo & Westgate, 2009) and seed composition responses to stress can vary among genotypes (Ortiz et al., 2022), the impacts of abiotic factors on seed composition are generally poorly understood. Elevated atmospheric CO_2_ has the potential to dilute nutritional elements in soybean seeds where yield is improved, and this response varies among genotypes (Digrado et al., 2024). While this quality reduction may be counteracted by other environmental factors such as increased temperatures (Köhler et al., 2019), this interaction has been explored in field conditions for very few genotypes.

### 4.2 Physiology and abiotic stress—Priorities (2024–2028)

Perhaps the greatest limitation in this thematic area is a lack of whole‐plant physiologists in the soybean community. The priority areas highlighted below require a mechanistic understanding of the physiology of soybean and a need to fill knowledge gaps through fundamental research questions. This foundational work is critical to advance to the stage of target gene identification and implementation of solutions through breeding and engineering.

A grand challenge for soybean physiologists will be to enable the development of varieties adapted for climate change, which encompasses new baseline environmental conditions as well as more frequent extreme weather stress. Many important genotype × environment questions could be investigated through coordinated, multi‐region field studies. Soybean presents some unique challenges for this kind of project, particularly with regard to photoperiod‐regulated development, but a community‐based curation of a diversity core set(s) available through Germplasm Resources Information Network (GRIN) for specific MGs and latitude ranges could nonetheless enable a large‐scale, coordinated project to evaluate the diversity in responses to uncontrolled environmental variation.

Controlled treatments are often required for abiotic stress research. At a minimum, this requires greenhouse or growth chamber space to conduct paired experiments. For some abiotic stresses, such as IDC, studies conducted in growth chambers and greenhouses identify the same QTLs as those grown in the field (Lin et al., 1998). Oftentimes, it is preferable to perform experiments in the field to maximize the relevance of experimental findings to production agriculture. Such work often requires expensive infrastructure to conduct replicated, well‐designed experiments, for example, movable rainout shelters, free‐air gas concentration enrichment, or open‐air canopy heating. The construction of all these infrastructures requires large initial investments, maintenance funding, and often a substantial amount of time for design and construction prior to conducting experiments. These factors can place field‐based abiotic stress and environmental physiology research outside the reach of many grant opportunities. Larger, collaborative teams may be able to secure sufficiently large grants to construct shared facilities, and this would be accessible to a limited number of scientists who could travel repeatedly to the site to take measurements, or to scientists who only required measurements at one point during a field season. It is crucial that funding agencies acknowledge the need for ongoing investment in experimental infrastructure to enable research that will protect soybean production in future climates.

To increase yield while improving seed quality, the soybean research community must work to identify bottlenecks and optimize carbon and nitrogen assimilation and partitioning to and within the seed. Many critical abiotic stress tolerance, productivity, and seed composition traits are genetically complex, which limits the utility of marker‐assisted breeding for soybean improvement. Integrative, physiological research is needed to illuminate the mechanisms underlying genotypic, environmental, and developmental variation in stress responses and central metabolic processes. Ecological physiology will be integral to these efforts, as the rhizosphere and shoot microbiomes are poorly understood and virtually untapped resources for potential crop improvement. Such research must have an integrative, whole‐plant perspective, since carbon and nutrient assimilation and partitioning are regulated via complex feedback between source and sink tissues and the environment. This work will be critical to identify target genes or processes for biotechnology‐based improvement and may also continue to illuminate new phenotyping strategies to aid breeding.

## 5 FUNCTIONAL GENOMICS

### 5.1 Functional genomics—Recent accomplishments

#### 5.1.1 Resources for standing variation and an expanded expression atlas

**FIGURE 2:**
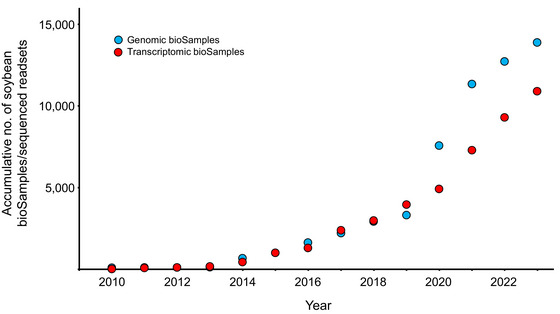
The accumulative total number of soybean bioSamples/sequenced readsets available for soybean genomic (blue spots) and transcriptomic (red spots) data in the NCBI SRA Database.

Assessing DNA sequence variation and gene expression variation in soybean has accelerated rapidly in the post‐genomic era, with a steep increase in the availability of such datasets in recent years (Figure 2). This has been a key development, as the driving force behind crop improvement is the introduction of functional genetic variation into the population. Recent advances in these areas are summarized below.

In traditional soybean breeding, novel genetic variation is usually derived from previously existing standing mutations. Recently, Zhang, Jiang, et al. (2022) consolidated, quality‐controlled, and analyzed whole‐genome raw‐sequencing reads of 1556 diverse soybean accessions and identified 32 million SNPs; each SNP was annotated using 30 structural and/or functional categories. The genomic diversity, geographic distribution, and population structure of 1500 soybean accessions and linkage equilibrium (LD) decay of wild soybean, landraces, and cultivars were determined. Interestingly, 30 SNPs per kilobase (kb) and an average of 12 nonsynonymous SNPs per gene are present in the 1500 accessions. Furthermore, Chan, Dietz, et al. (2023) recently analyzed single‐nucleotide variation in over 1000 soybean lines. This curated dataset was made available to the community for exploration through a web‐based tool (https://soykb.org/SoybeanAlleleCatalogTool/).

Large‐scale transcriptional analyses on public RNA‐seq and microarrays provide valuable targets for tissue‐specific research and help uncover broader biological patterns, and several large soybean transcriptomic studies have been submitted to NCBI in recent years (e.g., DeMers et al., 2021; Elmore et al., 2022; Kohlhase et al., 2021; Lu et al., 2023; Redekar et al., 2017; Sinha et al., 2023; Sreedasyam et al., 2023; Waldeck et al., 2017; Wang et al., 2019; Yung et al., 2022; Zhou et al., 2023; Zhu et al., 2022). Su et al. (2022) analyzed 5422 datasets representing a wide array of conditions including all major tissue types and developmental stages. The analysis helped to elucidate the dynamics of transcriptional regulation across this broad range of samples and experimental conditions. Expression networks based on developmental stage or tissue type were developed and published for thousands of cultivars (Almeida‐Silva et al., 2020; Su et al., 2022). Furthermore, a database hosting RNA‐seq data from 5481 samples is now available (Almeida‐Silva et al., 2023).

#### 5.1.2 Development of soybean mutant resources and other tools for functional genomics

The relatively low genetic diversity in soybean relative to other major crops limits the available phenotypic variation that can be exploited for gene discovery and crop improvement. One means to overcome this limitation is through induced mutagenesis, which is a relatively inexpensive and rapid approach to creating a large number of genetically diverse mutants. To this end, the soybean community has successfully developed large mutant populations in MG I, MG III, and MG VII cultivars that can serve the majority of US soybean researchers.

In recent years, efforts were made to further develop and characterize the fast neutron (FN) mutant populations at the University of Minnesota (UMN) (Bolon et al., 2014) and the University of Missouri (MU) (Stacey et al., 2016). The UMN population was developed in the M92‐220 cultivar (MG I; adapted to the northern United States) and consists of over 27,000 M2 families. The MU population was developed in the Wm82 cultivar (MG III; adapted to the central United States) and consists of over 10,000 M2 families. Additional FN populations, consisting of ∼2400 M2 families, were also developed in the high‐yielding elite genotypes G00‐3213 and G00‐3880 (MG VII, adapted to the southern United States) at the University of Georgia (UGA) (Prenger, Ostezan, et al., 2019). One advantage of FN mutagenesis is its ability to induce genetic deletions, which can be rapidly identified by comparative genomic hybridization (CGH), a rapid and cost‐effective method of identifying copy number variations (CNVs). CGH analysis of select mutants in the UMN and MU populations identified >500 Mb nonredundant deletions (>50% genome), of which ∼267 Mb (28.1% genome) are homozygous (https://soybase.org/projects/fn_mutants/). Forward and reverse genetic studies of FN mutants identified novel genes or new alleles involved in soybean protein/oil composition (Dobbels et al., 2017; Prenger, Ostezan, et al., 2019), seed size (Nguyen et al., 2021), nitrogen fixation (Nguyen et al., 2023), and timing of flowering and seed maturation (Dietz et al., 2021). Moreover, several seed composition mutants have been identified using forward genetic approaches to elucidate the genetic control of soybean seed development, particularly oil and protein production (Islam et al., 2020).

Soybean mutant populations have also been developed using chemical mutagenesis to induce genome‐wide SNPs. The soybean cultivar Forrest (MG V) and PI88788 were used to generate M2 populations using ethyl methanesulfonate (EMS) at Southern Illinois University (SIU). The SIU population consists of 4032 M2 lines with an estimated mutation density of ∼1/227 kb (Lakhssassi, Zhou, et al., 2021). A mutant library of the SIU population is available for high‐throughput TILLING (Targeting Induced Local Lesions in Genomes) by target capture sequencing technology, or TILLING‐by‐Sequencing. A population of over 8000 inbred Wm82 mutants was also developed using *N*‐methyl nitrosourea (NMU) at the USDA‐ARS in West Lafayette, IN. Screening of the EMS and NMU population using TILLING‐by‐Sequencing provided novel alleles for the modification of fatty acids and carbohydrates in soybean seeds (Lakhssassi, Lopes‐Caitar, et al., 2021; Thapa et al., 2019). An EMS population with 1820 lines was also created in JTN‐5203 (MG V) at Tennessee State University (Espina et al., 2018). Forward genetic screening of the EMS and NMU populations provided stable seed composition mutants to identify genes involved in oil and protein content (Hudson, 2022; Zhou et al., 2019).

Additionally, functional analyses have been facilitated by significant advances in tools such as virus‐induced gene silencing (VIGS) and virus‐mediated planta expression. The *bean pod mottle virus* (BPMV) has been optimized for VIGS and transient overexpression of genes of interest in soybean (Whitham et al., 2016), and this application allowed functional validation of resistance genes and mechanisms of defense against viral, fungal, and bacterial pathogens (e.g., Pedley et al., 2019; Tian et al., 2020; Tran et al., 2018; Wei et al., 2023). BPMV vectors have also been adapted to study ASR effectors through host‐induced silencing (Link, 2022). An alternative method that uses *cowpea severe mosaic virus* (CPSMV) as a vector was recently developed (Zaulda et al., 2022). Since CPSMV can infect soybean and *Nicotiana benthamiana*, viral constructs can be delivered initially to *N. benthamiana* for amplification through *Agrobacterium* infiltration, and the infected tissues can then be used to infect soybean. This approach eliminates the need for DNA bombardment. The CPSMV system has been optimized for silencing and expression of proteins in soybean and could facilitate high‐throughput functional analyses. Clustered Regularly Interspaced Short Palindromic Repeats (CRISPR), Transcription Activator‐Like Effector Nucleases (TALEN), and Zinc Finger Nucleases (ZFN) technologies have been adapted for use in soybean (reviewed in Xu et al., 2020), and they hold immense potential for functional analyses and crop improvement. However, few plant‐related traits have been targeted using these technologies (reviewed in Xu, Guo, et al., 2022), as the transformation bottleneck is still the main barrier to wider deployment of these genome editing options in soybean.

#### 5.1.3 Elucidation of key genes and pathways governing important soybean traits

Rapid and continued development of various genomic resources and tools has led to the discovery of numerous QTLs controlling various traits of agronomic importance, including seed composition and size, flowering time/maturity, plant architecture, efficiencies for nodulation and symbiotic nitrogen fixation, and domestication‐related traits (DRTs). Additionally, key genes responsible for several major QTLs have been identified and functionally characterized, including elucidation of the underlying genetic pathways for some traits.

Seed composition traits, such as protein and oil content, are quantitative and are generally modulated by many minor QTLs; however, major QTLs with large effects have also been discovered. As seed protein and oil content are usually negatively correlated (Chaudhary et al., 2015), these QTLs often show pleiotropic effects on both seed oil and protein content as well as additional seed traits, such as seed size and weight. The QTL with the largest effect on seed composition is probably the *Bloom1* (*B1*) locus controlling seed coat bloom (Zhang et al., 2018). The loss‐of‐function mutation at this locus not only led to the domestication transition from the “bloom” to “no‐bloom” phenotypes but was also responsible for elevating seed oil content in cultivated soybean. A few SWEET (Sugars Will Eventually bE Transported) genes encoding sugar transporters, including two functionally redundant homeologs, *GmSWEET10a* and *GmSWEET10b* (Miao et al., 2020; Wang et al., 2020), and *GmSWEET39* (Zhang et al., 2020), have been found to be associated with high seed oil content and low seed protein content. Because these are involved in the allocation of sugar from the seed coat to the filial embryo, they have positive effects on seed size (Wang et al., 2020; Zhang et al., 2020). Goettel et al. (2022) and Fliege et al. (2022) recently identified a CCT‐domain‐containing gene (also known as *POWR1*), which underlies high seed protein content, low seed oil content, and low seed weight through regulating lipid metabolism and nutrient transport genes. In addition, four triacylglycerol lipase‐encoding genes (*GmSDP1‐1*, *GmSDP1‐2*, *GmSDP1‐3*, and *GmSDP1‐4*) (Kanai et al., 2019), an oleosin protein‐encoding gene (*GmOLEO1*) (Zhang, Zhang, et al., 2019), and several TF‐encoding genes (*GmWRI1a* and *GmWRI1b* [the orthologs of Arabidopsis *WRINKLED1*], *GmLEC2* [the ortholog of Arabidopsis *LEAFY COTYLEDON2*], and *GmABI3* [the ortholog of *ABSCISIC ACID INSENSITIVE3*]) have been found to be involved in fatty acid biosynthesis and seed oil content (Chen et al., 2018; Guo et al., 2020; Zhang et al., 2018).

Grain yield is one of the most important and complex traits that has been targeted in soybean breeding. The major QTLs and natural variations directly responsible for high yield are rare. Nevertheless, genes underlying key yield component traits, such as the total seed number per pod, seed size and weight, as well as flowering time/maturity and plant architectural traits that largely affect soybean yield potential, have been identified.

Most of the flowering time/maturity genes in soybean are orthologous/homologous to previously identified flowering‐related genes in Arabidopsis (Zhang, Liu, et al., 2022). In some cases, however, they have evolved new functions. For example, the *J* gene in soybean (gene model Glyma.04G050200), the homolog of Arabidopsis *Early Flowering 3* (*ELF3*), underlies the long‐juvenile trait and enables soybean to adapt to tropical regions (Lu et al., 2017; Yue et al., 2017). Similar to those observed in Arabidopsis, many of the flowering genes in soybean interact with each other to modulate their functions (Zhang, Liu, et al., 2022).

Seed size is generally associated with seed weight and is controlled by multiple QTLs (Swarm et al., 2019). The QTLs with the largest effects on seed size are those associated with the domestication transition. In addition to *POWR1* (Fliege et al., 2022; Goettel et al., 2022), which has a pleiotropic effect on seed weight, a gene encoding a phosphatase (PP2C‐1) was targeted for selection during domestication and contributed to seed size increase in cultivated soybean through the brassinosteroid signaling pathway (Lu et al., 2017). Screening an FN mutant population identified GmKIX8‐1, a putative kinase‐inducible domain‐interacting protein, which regulates seed size (Nguyen et al., 2021). *GmKIX8‐1* is located within the major seed weight QTL qSw17‐1, previously mapped using several biparental populations (Fujii et al., 2018; Liu et al., 2022; Liu, Yan, et al., 2018).

Plant architectural traits, such as plant height, stem growth habit, branch angle and number, and leaf petiole angle, are critical for plant productivity and/or grain yield. Wang, Li, et al. (2021) reported that CNV of *gibberellin 2‐oxidase 8* genes is associated with changes in trailing growth and shoot length associated with soybean domestication. A major QTL (*GmBa1*) modulating branch angle was identified by linkage analysis using biparental mapping populations with and without pedigree from wild soybean (Clark et al., 2022; Virdi et al., 2023). The *GmBa1* region overlaps with the QTL region underlying phenotypic variation in canopy coverage, suggesting that branch angle is a major determinant of canopy structure. Shim et al. (2019) identified four QTLs associated with branch number, including a region harboring the ortholog of the Arabidopsis *BRANCHED1* (*BRC1*) gene, which acts inside axillary buds as an integrator of branching signals to control lateral branch development. Recently, a GWAS analysis suggested that *Dt2*, which interacts with *Dt1* to produce a semi‐determinate stem growth habit (Liu et al., 2016), is a key determinant of branch number (Liang et al., 2022). However, because a set of diverse varieties adapted to various eco‐regions were chosen and phenotyped in the same environments, the effect of *Dt2* on branch number could have been overestimated. Zhang, Wang, et al. (2019) found that *Dt2* was also associated with plant sensitivity to water deficiency by interacting with genes associated with stomatal activities and genes responsive to drought stress. Gao et al. (2017) discovered that a gene encoding an APC8‐like protein, *GmILPA1*, controls leaf petiole angle by screening a soybean EMS‐mutant population, but no genetic variation responsible for phenotypic variation has been identified in the natural populations.

Nodulation and symbiotic nitrogen fixation processes are vital for soybean plant development and growth. Through forward genetic, genomic, transcriptomic, and translatomic analyses, numerous genes and regulatory elements, such as mobile microRNAs, involved in genetic pathways/networks underlying these processes have been identified (Liu et al., 2023; Roy et al., 2020; Zhang, Su, et al., 2021). Different types of shoot–root mobile elements, including proteins (Li, Zhou, et al., 2022; Wang, Guo, et al., 2021), small peptides (Kereszt et al., 2018), and microRNAs (Okuma et al., 2020), have been reported to regulate nodulation and/or symbiotic nitrogen fixation. In addition, rhizobial tRNA‐derived fragments were found to enable the regulation of soybean genes to facilitate nodulation (Ren et al., 2019).

The domestication of soybean is a complex process of artificial selection for a suite of favorable traits (Sedivy et al., 2017). Using two large recombinant inbred line populations derived from crosses between a soybean cultivar and each of two *G. soja* accessions, over 100 QTLs associated with various DRTs have been identified and mapped to chromosomal regions (Swarm et al., 2019). In addition to several genes underlying key DRTs described earlier (Sedivy et al., 2017), *B1* modulating seed coat bloom (Zhang et al., 2018) and *POWR1* and *GmKIX8‐1* regulating seed compositions and size (Fliege et al., 2022; Goettel et al., 2022; Nguyen et al., 2021) were targeted for selection through soybean domestication.

### 5.2 Functional genomics—Priorities (2024–2028)

#### 5.2.1 Develop sequence‐indexed resources for mutant populations

Comprehensive mutant populations using a variety of different approaches are critical to address the need to decipher soybean gene function using forward and reverse genetics. Large populations of loss‐of‐function soybean mutants were developed for the soybean community through FN and chemical mutagenesis. The FN populations were genotyped mainly through CGH, a method that detects large insertions and deletions (indels) ranging from ∼2 Kb up to >8 Mb. However, FN can also induce small indels that can result in frameshift mutations (Wyant et al., 2022). Therefore, to maximize the capture of induced indels, it is important to genotype the FN mutants, especially those already selected for important agronomic traits, using long‐read sequencing technology. The EMS and NMU populations were mostly utilized by the soybean community for TILLING, which requires a substantial amount of work to identify mutants for each gene of interest. Whole‐genome sequencing of these mutants would therefore allow the construction of an online in silico TILLING system. Lastly, continued efforts are needed to create a large population of gain‐of‐function mutants through activation tagging that can uncover genes that function redundantly, are compensated by alternative metabolic/regulatory pathways, or are associated with lethality.

To enhance the use of soybean mutant resources, we prioritize the following outcomes: (1) development of a sequence‐indexed mutant database in which most soybean genes are knocked out or downregulated; (2) availability of genome sequences for at least 30 soybean mutants with agronomically important traits, for example, seed composition, plant architecture, and biotic/abiotic stress; and (3) generation of an abundant resource of enhancer trap lines that activate soybean genes.

#### 5.2.2 Establish a community seed repository for soybean functional mutant collections

The soybean community has mutant populations that cost millions of dollars to develop. Considerable resources were also invested in screening the mutants for reverse and forward genetic studies. So far, these mutants are being curated by the researchers who developed them, which is not sustainable. Establishment of a genetic repository and distribution center for soybean mutants and transgenic lines was identified as a primary goal in the two prior strategic plans. USDA/ARS allocated funds to the USDA Soybean Germplasm Collection to establish a repository to house a collection of FN seed lots. However, individual labs/researchers would still be responsible for replenishing seed stocks to keep mutant seeds viable.

Given its importance, funds should be allocated to establish and maintain a repository of mutant and transgenic seed lots, particularly those that have been genotypically and/or phenotypically characterized. This need will be more critical in the next 5 years as the community will continue to characterize existing mutants and generate more gene‐edited soybean lines. The soybean community must address the need to establish long‐term storage of seeds through cryo‐preservation.

Anticipated outcomes from this effort will include the following: (1) A permanent soybean seed repository is established. (2) A standardized methodology for depositing mutant/transgenic lines to soybean seed repository will be developed. (3) An online ordering system for mutant/transgenic lines will be created. (4) A cryo‐preservation protocol and long‐term seed storage repository will be established.

#### 5.2.3 Obtain additional “omics” data to understand molecular mechanisms governing important traits

A majority of the available gene expression atlases were generated to determine tissue‐specific gene expression in a given soybean cultivar. To better understand the molecular mechanisms underlying important traits, gene expression datasets to identify differentially expressed genes and proteins between disparate genotypes are needed. For example, identifying differentially expressed genes/proteins between seed composition mutants and their corresponding wildtype parents during seed development can identify critical genes, gene networks, and metabolic pathways that can be exploited for seed quality improvement. Of note is the availability of seed storage mutants for functional genomics studies elucidating the molecular mechanisms underlying protein rebalancing (Herman, 2014). There is also a need to generate additional “omics” data to understand the molecular and physiological mechanisms governing biotic and abiotic responses in soybean. For example, an initial study (Kohlhase et al., 2021) examined iron stress responses across 18 soybean genotypes with differing iron stress tolerances, demonstrating little overlap in gene expression. In addition, this study highlighted differences in soybean iron stress responses relative to model species. Likewise, “omics” datasets comparing disease‐resistant and susceptible soybean genotypes during pathogen challenges need to be expanded. Lastly, although field‐grown plants are exposed to multiple abiotic stresses, most functional genomics studies have focused on single stressors such as heat or drought. These studies should be extended to multiple abiotic stressors and genotypes to better understand how soybean responds to multiple climate‐related patterns, for example, high heat and drought. For example, recent work has examined soybean gene expression responses to repeated and multiple stresses (O'Rourke & Graham, 2021; O'Rourke et al., 2020). In this study, repeated stress and multiple stress resulted in novel gene expression changes not detected in a single stress experiment.

Anticipated outcomes for these efforts include the following: (1) Seed developmental transcriptome and proteome datasets comparing seed composition mutants and corresponding wild‐type parents are generated. (2) Transcriptome datasets comparing disease‐resistant and susceptible genotypes upon pathogen challenge are generated. (3) Transcriptome datasets under single and multiple stress conditions are generated.

#### 5.2.4 Develop soybean protein–protein interaction databases

To perform processes and functions within a cell, most proteins interact with others, forming protein complexes and molecular machines. Therefore, characterization of protein–protein interactions (PPIs) can facilitate the understanding of proteins and their molecular functions. Experimental data for PPIs are primarily generated using yeast two‐hybrid and co‐immunoprecipitation coupled mass‐spectrometry techniques, which are expensive, time consuming, and labor intensive. These methods are primarily useful for the validation of a few PPI pairs but not for genome‐wide PPI predictions. The advancements in high‐throughput computational approaches have made large‐scale prediction of PPIs relatively rapid and efficient. So far, there are no available PPI databases for soybean. A large volume of information exists on soybean nodulation (and in the related species *Medicago truncatula* and *Lotus japonicus*) to enable the development of robust PPI databases for nodulation. Although not as extensive as nodulation, “omics” data are available (and will be further developed) on seed development, soybean–pathogen interactions, and abiotic stress.

The primary anticipated outcome from this effort will be the development of PPI databases for soybean root nodule development, seed development, and biotic/abiotic stress responses.

#### 5.2.5 Develop single‐cell “omics” datasets in soybean

The soybean single‐cell transcriptome atlas is an upcoming resource that reveals the patterns of expression of each gene in each cell composing the soybean plant (i.e., root, nodule, true leaf, trifoliate leaf, shoot apical meristem, floral buds, green pods, and seeds at different developmental stages of the soybean plant). As a result, the transcriptome of 120,000 nuclei has been analyzed, leading to the identification of 157 different cell clusters and the detection of the expression of 88.5% of soybean protein‐coding genes (Cervantes‐Perez et al., 2024). The open sharing of the soybean single‐cell transcriptome atlas will allow soybean scientists to precisely analyze the activity of their genes of interest and to identify soybean cell‐type‐specific promoter sequences to contribute to the development of new genetic engineering strategies to improve soybean biology.

#### 5.2.6 Upgrade existing online repositories for new functional genomics datasets

A large body of functional genomics datasets have been developed and require a permanent repository with sufficient and stable funding. In the next 5 years, the USDA/ARS SoyBase team intends to integrate single‐cell RNA (scRNA) experimental data into SoyBase to provide insight into the tissue and treatment expression patterns of the transcriptome and individual genes. During this time, it is reasonable to assume that the number of large‐scale phenotype experiments with genotype data will increase. SoyBase will need to effectively organize existing datasets to enable community access. SoyBase will begin to collect and display multiple omics datasets such as ionomic, metabolomic, and interactomic data. Finally, SoyBase will be continually updating the composite genetic map with biparental QTLs and the physical map with GWAS QTLs. Visual display of these emerging data types will be important, and the SoyBase team will continuously evaluate software solutions to serve the widest possible user base.

The key anticipated outcome from this effort will be the establishment of a sustainable soybean data repository, with the flexibility to incorporate new data types as they become widely used by the community.

## 6 BIOTECHNOLOGY

### 6.1 Biotechnology—Recent accomplishments

**FIGURE 3:**
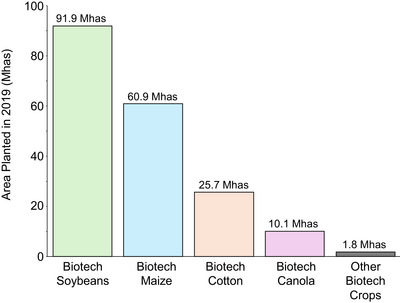
Global land area planted to biotech crops in 2019, presented in million hectares (Mhas) (ISAAA, 2019). The category “Other Biotech Crops” includes biotech sugar beets, potatoes, apples, squash, papaya, and brinjal/eggplant.

During the last survey of biotech crops, conducted in 2019, soybean accounted for almost half the global land area planted for biotech crops (ISAAA, 2019) (Figure 3). Transgenic alleles deployed in soybean impart multiple types of herbicide tolerance, insect resistance, and improved oil quality. In addition, the first gene‐edited allele to reach the market in soybean also translates to improved oil quality.

Adding novel genetic variation, consisting of transgenic and/or edited alleles into soybean, like other crops, requires transformation technology. Soybean transformation has undergone several refinements over the past three decades, leading to incremental improvements in the technology (Xu, Guo, et al., 2022), but bottlenecks remain. As is the case with most other crops, soybean transformation is labor intensive, with high genotypic variability, and thus a challenge to meet the current demands from the research and the breeding communities (Altpeter et al., 2016).

The goal is to continuously improve our ability to add novel genetic variation to soybean via transgenic and/or edited alleles, with efficiency gains measured by economic and time metrics. Addressing this goal will require advances in technology coupled with engineering tools.

### 6.2 Biotechnology—Priorities (2024–2028)

Plant transformation has two requirements. First, DNA must be delivered into the cell, and second, that cell must be able to form a fertile plant. Transformation efficiency is then defined as the multiplicative product of the probability that a soybean cell has the competence for both of these events. Thus, investigations addressing improvements in either or both parameters will lead to improved transformation efficiency.

#### 6.2.1 Better DNA delivery

Historically, the two main ways to engineer soybean are microprojectile bombardment and *Agrobacterium*‐mediated transformation (AMT) (Parrott & Clemente, 2004). The popularity of AMT has increased in recent years due to its perceived advantages in producing quality events (i.e., single‐copy insertions with no rearrangements for vector backbones), as compared to the direct DNA delivery method of microprojectile bombardment.

The current use of AMT depends on strains derived from just two *Agrobacterium* strains—Ach5 (octopine strain) and C58 (nopaline strain) (De Saeger et al., 2021). Hence, there is potential to enhance transformation by exploring other strains (Altpeter et al., 2016). These could be more virulent on soybean (e.g., Torisky et al., 1997) or more efficient by evading the soybean immune system, thus expanding the range of genotypes amenable to AMT. The potential of this approach has been recently demonstrated by the use of *Ochrobactrum haywardense* H1 (Cho et al., 2022) to replace *Agrobacterium* for soybean transformation.

The usefulness of strains used for AMT can be augmented through a series of mutations to create stealth (i.e., the ability to evade the soybean immune system) strains, RecA mutants to prevent plasmid scrambling in the bacterium, and the use of *Pseudomonas* Type III secretion systems for protein delivery (De Saeger et al., 2021). Auxotrophic strains increase efficiency and reduce the adverse effects of antibiotics on the explant tissue, and the first auxotrophs of the modern era are becoming available (Aliu et al., 2024; Prías‐Blanco et al., 2022).

Finally, an assortment of ternary vectors (van der Fits et al., 2000) designed to enhance transgene delivery, particularly of multiple‐gene‐containing constructs, needs to be available.

#### 6.2.2 Narrowing the gap with conventional breeding

Ideally, the ability to add novel genetic variation to soybean will have a reduced genotype dependency, thereby strengthening editing and transformation as a means to complement soybean breeding programs and for use in research and development activities. Editing elite genotypes would bypass backcrossing altogether. The inability to use elite genotypes is particularly acute in the public sector.

A key step toward expanding soybean transformation genotype flexibility is to be able to bypass or mitigate the tissue culture component, as is being explored in other species (Cody et al., 2023). The use of morphogenic genes is expected to be particularly useful to meet this goal.

Tissue culture remains very labor intensive, and some steps in transformation, such as the selection of the right type of tissue, still require a judgment call by the practitioner. Hence, complementary technologies to increase the efficiency of soybean tissue culture and transformation could play a key role in improving efficiency. The solution lies in the application of robotics equipped with machine vision and AI and its application to soybean improvement (Hesami & Jones, 2020).

#### 6.2.3 Engineering multiple traits/biochemical pathways

The breeding trend is toward stacking multiple traits/biochemical pathways into elite varieties. Such multi‐stack transgenic and/or edited alleles will need to be introgressed into the appropriate genetic backgrounds. Currently, most transgenes deployed are in breeding stacks—that is, transgenes are individually engineered, and the resulting plants are then crossed together. The issue is that the number of independently segregating traits is exceeding the ability of breeders to handle them. Thus, the goal is to use molecular stacks—that is, the use of multiple cisgenes and transgenes in one single vector. This approach is also expected to enable metabolic engineering, the production of bioproducts and value‐added products, and to facilitate the application of synthetic biology.

**FIGURE 4:**
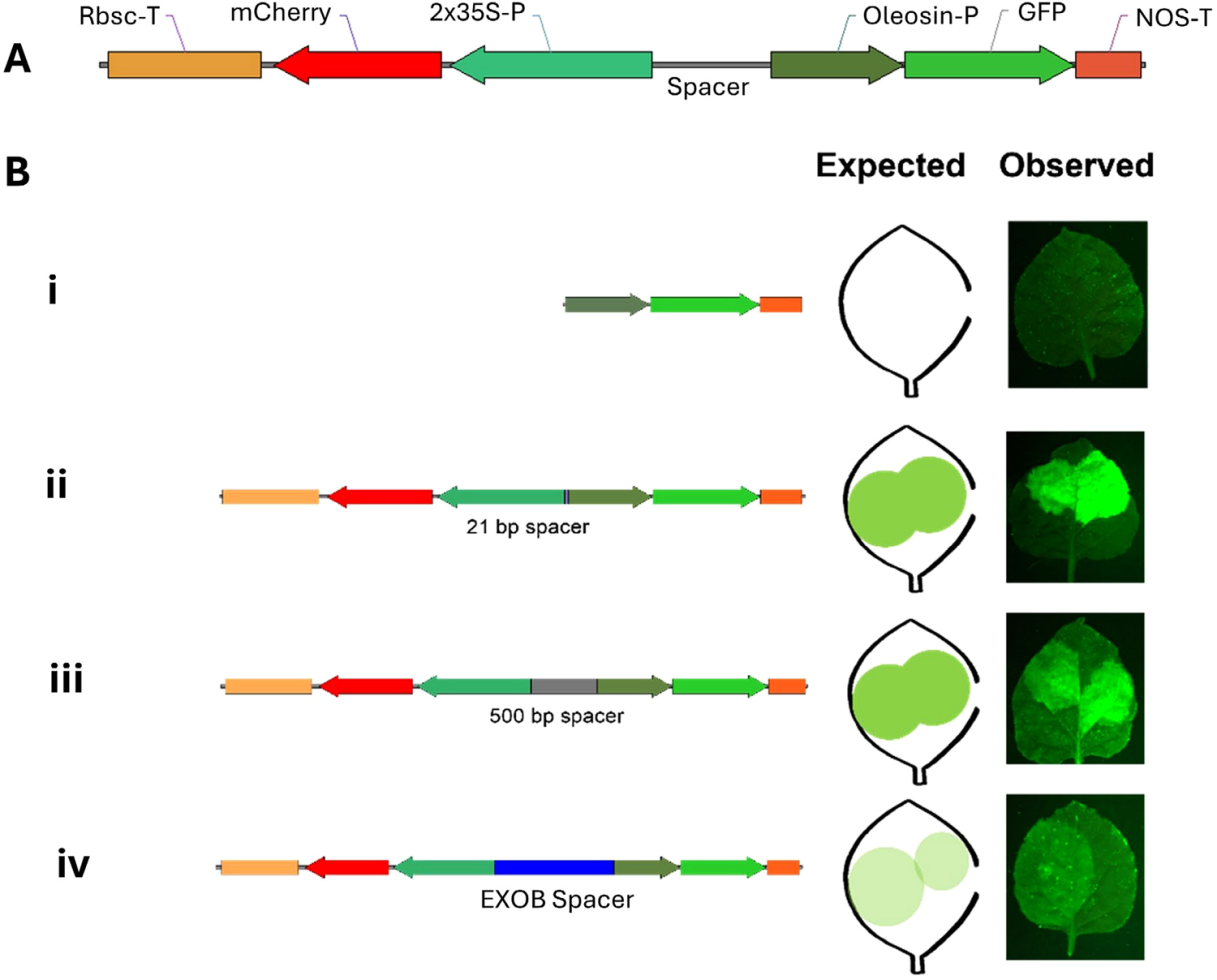
Interference between enhancers and promoters of transgene constructs can lead to unwanted expression patterns. (A) Map of construct used to test the function of putative insulator sequence. (B) Agroinfiltration of *Nicotiana benthamiana*. (i) Green fluorescent protein (GFP) driven by the seed‐specific oleosin promoter is not expressed in leaves, unless (ii) ectopic expression from an enhancer in an adjacent 35S promoter activates it. Adding (ii) 21 bp or (iii) even 500 bp is not enough to attenuate the ectopic expression, but adding the EXOB insulator from phage lambda (iv) does attenuate it. Data provided by the Wayne Parrott lab.

Early impediments for the assembly of long transgene constructs have been overcome (e.g., Collier et al., 2018; Lampropoulos et al., 2013), and ternary vectors can facilitate their delivery through AMT. However, there are still major knowledge gaps. First, more tissue‐specific or synthetically designed promoters (Belcher et al., 2020) are needed, which in turn requires a greater understanding of cis‐regulatory regions and TF binding sites. Second, better coordinated expression of the transgenes is needed, as illustrated in Figure 4.

In multigene constructs, enhancers in some promoters will alter the expression patterns of nearby genes. The use of insulators (transcription‐blocking sequences) restores independent control to each transgene, as can be seen below when the EXOB insulator sequence is used as a spacer. Currently, very few such sequences are known that work in plants (e.g., Jiang et al., 2022), and additional discovery is needed. Since the action of such transcription blockers can be context specific, it remains to be seen if their function can be validated in soybean.

#### 6.2.4 Enabling CRISPR

The use of CRISPR associated with Cas9 and Cas12 endonucleases has become widespread as a method to knock out genes for functional genetics studies or to eliminate undesirable traits (Zhu, Li, et al., 2020). However, CRISPR is capable of far more useful types of gene editing, which have yet to be fully enabled in soybean (Bao et al., 2020). These include:

- Enabling prime editing and base editing for small, targeted changes to alleles;
- Enabling Cas‐mediated gene silencing/activation, as recently demonstrated (Pan & Qi, 2023);
- Phloem‐dispersible guide RNAs;
- Transposase‐assisted target‐site integration, as recently demonstrated (Liu et al., 2024);
- Site‐directed knock‐in technology.

Transposase‐assisted target‐site integration and site‐directed knock‐in technology could be particularly useful for the development of platforms that allow for site‐specific integration of transgenes into chromosomes. These integration sites may be “safe harbors” for transgenes, such that they do not mutate or affect the expression of neighboring genes (Gao et al., 2020). This recombination‐based technology, originally developed for maize, has yet to be ported to soybean.

#### 6.2.5 Enabling technologies for advanced breeding

Three major issues were identified that have potential biotechnological solutions.

- The first is the production of doubled haploids. Recent breakthroughs in the molecular understanding of haploid production are enabling haploid production in monocot species, and this understanding has recently been extended to dicots (Zhong et al., 2020), meaning the technology is ready to extend to soybean.
- Second is pollination control for hybrid seed production. Canola (*Brassica napus* subsp. *napus*) has long used genetically engineered male sterility for hybrid seed production, and the system has been adapted for use with Indian mustard (*Brassica juncea* L.) (Chand et al., 2018). It may be possible to implement it in soybean with additional modifications.
- Finally, gene flow mitigation was raised as a topic to help reduce regulatory concerns (Clark & Maselko, 2020).

#### 6.2.6 Specialty soybean

**FIGURE 5:**
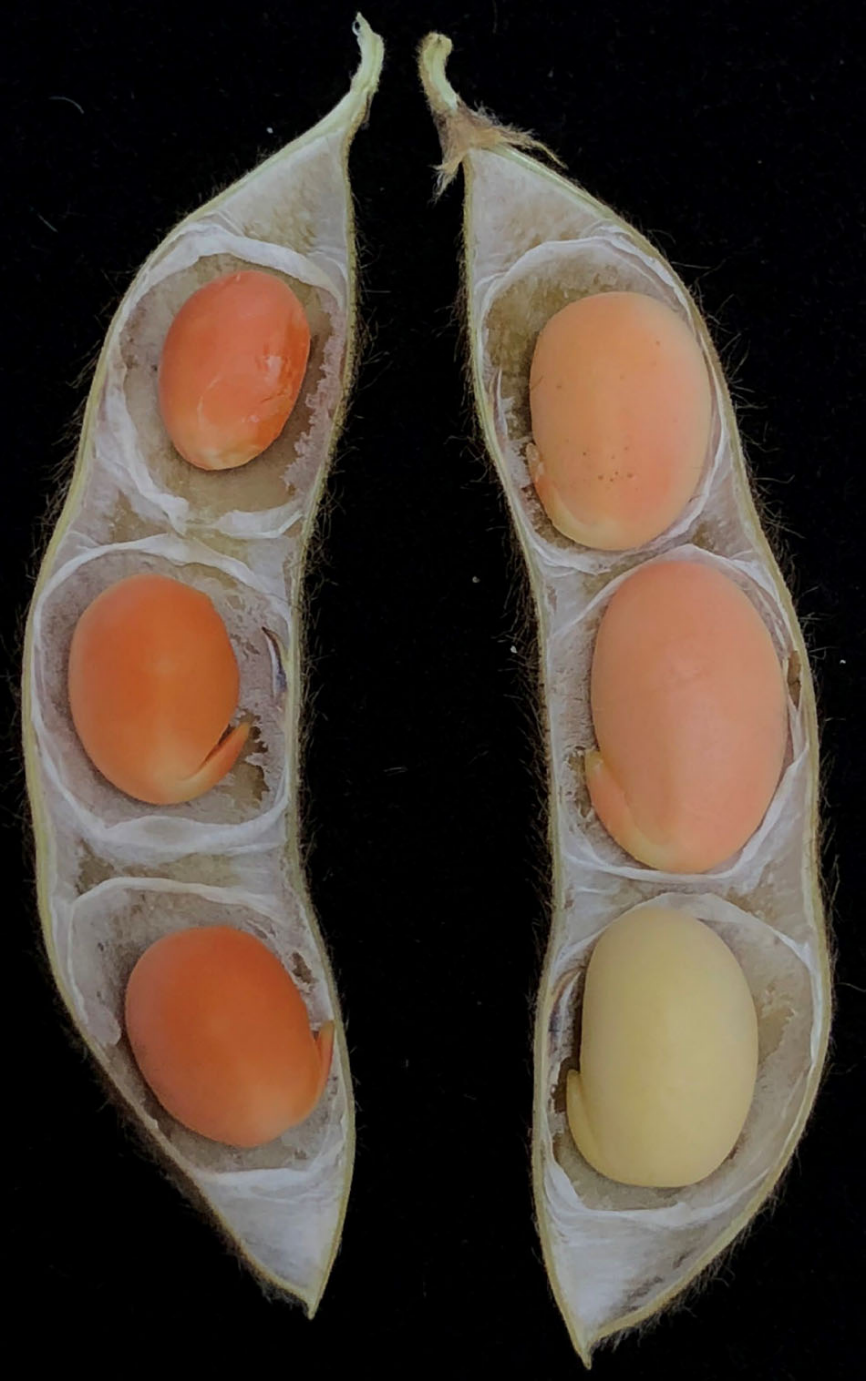
Astaxanthin production in soybean. Image courtesy of Ed Cahoon and Tom Clemente.

Soybean is still considered a new crop in that it has become a commodity over the past half‐century. Historically, commodity crops spin off specialty varieties over time. For example, the production of high‐oleic soybean varieties is increasing (Clemente & Cahoon, 2009). However, there are other emerging markets and uses, and chief among these is aquaculture, which is transitioning to land‐based diets (Herman & Schmidt, 2016). Soybean optimized via biotechnology to feed fish needs to produce long‐chain omega‐3 fatty acids, astaxanthin, and taurine (Park et al., 2017) (Figure 5). In addition, soybeans with altered composition can help expand the market for the crop through the development of more direct food product offerings to the consumer, such as plant‐based meat substitutes.

#### 6.2.7 Microbial biotechnology

Soybean is able to control several of the pathogens and beneficial microbes in its rhizosphere that are associated with plant health and productivity, though a foundational understanding of such plant–microbe interactions is still lacking. There are diverse soybean root exudates (Sugiyama, 2019) and a suite of genes in soybean and its symbionts that select for certain microbes or strains (Zhang, Wang, et al., 2021; Zhao et al., 2018). With a greater knowledge of these, it will be possible to develop “designer” soybeans that only nodulate with the most desirable strains available in their rhizosphere.

#### 6.2.8 Risk assessment support

Regulatory barriers continue to delay the approval and deployment of edited and transgenic soybean around the world. In large measure, there is a lack of understanding of plant genomes around regulatory agencies, leading to the perception that SNPs, indels, and other changes can make food harmful to humans (Kessler et al., 1992). The solution has been to provide context on standing variation in the soybean genome (Anderson et al., 2016) and plant genomes in general (Graham et al., 2020). Such context makes it possible to measure the potential harmfulness of a new SNP/indel compared to similar changes found in the standing variation of the genome. Accordingly, assembling a catalog of SNPs and structural variation in the main soybean germplasm pool would be extraordinarily helpful.

#### 6.2.9 Stewardship

The advent of CRISPR has democratized biotechnology, and the use of morphogenic genes for regeneration from tissue culture is expanding the range of crops and genotypes amenable to editing and transformation. Given the relaxation of USDA rules on biotechnology, some leading scientists believe that regulatory and public concerns have been overcome. However, gene flow and seed mixtures can still occur, and while highly unlikely that such an outcome will lead to environmental and/or food safety issues, practitioners of editing and transformation must be cognizant of proper stewardship practices to mitigate the probability of market/trade shutdowns.

Far less dramatic outcomes are also possible. However, any negative publicity will alienate the public, contribute toward a negative perspective on science and technology, and increase the time needed for these technologies to become mainstream. Therefore, it is incumbent upon all developers and users of edited and transgenic soybean to incorporate simple measures into their standard operating procedures that will help prevent the inadvertent release of transgenes/edited alleles into the soybean commodity crop.

#### 6.2.10 Germplasm repository

Soybean biotechnology is expensive; yet, soybean seed viability is relatively short, and the necessary infrastructure for proper storage and distribution of seed is lacking. As described in Section 5.2 above, the soybean community has no avenue to maintain soybean germplasm that carries novel genetic variation, including transgenic and edited alleles, along with unique mapping populations. Hence, the establishment of a soybean biotechnology germplasm repository is a critical short‐ and long‐term need. Such a repository will need to include a place to deposit edited and/or transgenic alleles, along with a coordinated network for grow‐outs of seed.

#### 6.2.11 Recruiting the next generation

Many of the original pioneers of soybean transformation have already retired. With time catching up to most of the remaining scientists with soybean transformation expertise, it is imperative to train the next generation before the remaining expertise and experience in soybean transformation technologies are lost. The urgency is immediate, and the need is critical.

This training must include experience in methodologies of soybean transformation, with an emphasis on the theory and practice of plant tissue culture, regeneration, and the underlying biology of transformation tools. This broad training approach will enable the soybean community to strengthen the Science, Technology, Engineering, and Mathematics (STEM) workforce with competence in plant transformation.

The biggest obstacle to training graduate students in a biological STEM field is funding. These costs include stipends, supplies, and indirect costs, totaling hundreds of thousands of US dollars over the course of graduate training. Since many of the traditional students in the United States are unaware of career opportunities related to plant science and agriculture in general, it is essential that the pool of STEM learners be broad to maximize the likelihood of attracting and developing a qualified workforce in this space.

#### 6.2.12 Conclusion

In 2024, adding novel genetic variation (whether transgenic and/or edited alleles) in soybean has become an important tool to complement breeding and functional genomics programs. As the technology evolves into true synthetic biology, the current products that are already commercialized or in the pipeline will one day be seen as comparatively simple. The advent of synthetic biology has the potential to create “designer” soybeans—tailored to produce value‐added products, adapt to changing environments, or serve new purposes (Wurtzel et al., 2019), and thus help fuel the emerging bioeconomy.

The priorities listed above are necessary to continue to meet the needs of the breeding and research communities. At the same time, these goals will create the infrastructure needed to start deploying synthetic biology within soybean.

## 7 GENOMIC RESOURCES AND DATASETS

Soybean was domesticated from a wild ancestor *Glycine soja* (Siebold & Zucc.) in East Asia, and domestication led to a significant loss of genetic diversity in cultivated soybean (Hyten et al., 2006). Genetic bottlenecks from artificial selection have significantly impacted modern soybean improvement capacities. In North America, fewer than 20 East Asian soybean landrace ancestors formed the foundational genetic basis for US soybean variety development (Gizlice et al., 1994). Rare alleles of soybean genes are especially abundant in accessions of *G. soja* (Chan, Dietz, et al., 2023; Hyten et al., 2006; Viana et al., 2022). The National Plant Germplasm System‐Germplasm Resources Information Network (NPGS‐GRIN) collection houses approximately 1500 accessions of *G. soja*. The *G. max* collection consists of over 20,000 available soybean accessions, including collected landraces and improved cultivars developed by intentional breeding. *Glycine max* and *G. soja* species are readily cross‐compatible. NPGS‐GRIN provides seeds to soybean researchers and stakeholders for requested accessions in the germplasm collection.

### 7.1 Genomic resources and datasets—Recent accomplishments

In recent years, several novel *G. max* reference genomes have been generated, two *G. soja* reference genomes have been released, and a pan‐genome analysis utilizing seven *G. soja* genomes has been conducted (Li et al., 2014; Shen et al., 2018; Valliyodan et al., 2019; Xie et al., 2019). There is also a pan‐genome analysis of wild and cultivated soybeans that skim‐sequenced 2898 accessions and created draft reference genomes for 26 representative accessions—though most of these accessions are not readily available outside China (Liu et al., 2020). Recently, near‐gapless reference genomes of soybean have been released (Espina et al., 2024; Garg et al., 2023; Wang, Zhang, et al., 2023; Zhang et al., 2023), with some differences noted between Wm82 assemblies (Espina et al., 2024). The number of *G. soja* and *G. max* protein‐encoding gene models has fluctuated markedly among the different genome assemblies released in recent years, ranging from ∼47,000 to nearly 60,000 (Table 2). This variation may be more readily attributed to differences in annotation pipelines used among research groups, rather than biological differences.

**TABLE 2:** Number of gene models in a subset of assemblies and annotations of different *Glycine max* and *Glycine soja* accessions generated in recent years (in chronological order by year of publication).

Species	Gene models	Genotype/version	Reference
*G. max*	48,387	Wm82.a6	Espina et al. (2024)
*G. max*	52,783	Fiskeby III	Espina et al. (2024)
*G. max*	55,498	Wm82‐NJAU	Wang, Zhang, et al. (2023)
*G. max*	50,564	Zhonghuang 13	Zhang et al. (2023)
*G. max*	58,287	Wm82.a5	Garg et al. (2023)
*G. max*	56,725	Lee	Garg et al. (2023)
*G. max* and *G. soja*	54,405–59,588	Various	Liu et al. (2020)
*G. max*	52,872	Wm82.a4	Valliyodan et al. (2019)
*G. max*	47,649	Lee	Valliyodan et al. (2019)
*G. soja*	46,969	PI 483463	Valliyodan et al. 2019)
*G. soja*	55,539	W05	Xie et al. (2019)
*G. max*	52,051	Zhonghuang 13	Shen et al. (2018)

There have been numerous soybean studies featuring large resequencing datasets in recent years (Bayer et al., 2022; Fang et al., 2017; Kim, Lozano, et al., 2021; Kou et al., 2022; Li et al., 2020; Li, Qin, et al., 2023; Lu et al., 2020; Qi et al., 2023; Valliyodan et al., 2021; Zhou et al., 2015). Among resequencing studies, soybean polymorphism number depends on the dataset; in the Liu et al. study, over 30 million quality‐vetted variant positions were identified (Liu et al., 2020; Valliyodan et al., 2021, 2016).

Significant resources were devoted to organizing and analyzing the rapidly increasing volume of genomic data for soybean accessions available as raw sequence reads. This large amount of publicly available data for so many lines has enabled deep analysis of potentially impactful new alleles by both public and private researchers. Additionally, as data became available electronically, deep analyses became possible without access to the germplasm itself.

### 7.2 Genomic resources and datasets—Priorities (2024–2028)

#### 7.2.1 Develop a comprehensive pangenome reflecting the diversity of soybean

As the number of distinct complete genome sequences for multiple cultivars of soybean and other species increased, it became clear that single reference genomes do not represent the diversity within a species. This is particularly evident when examining functions such as biotic stress defense response, as it is known that gene duplication and differentiation are critical to maintaining pest defense responses. Pan‐genomes, which combine multiple genome sequences and are particularly useful for examining pests and diseases that impact soybean production, go a long way toward addressing this issue.

Considerable progress toward a soybean pangenome has been made via sequencing of diverse accessions, mostly using short‐read technologies. However, these results rely on reference‐based variant calling and do not contain extensive data on structural variation, which is now known to be important for several key agronomic traits in soybean. In addition, many published efforts do not address US germplasm and/or utilize lines that are not publicly available to US researchers. A pan‐genome based on high‐quality, long‐read technology and focused on the diversity within the USDA Soybean Germplasm collection has been approved by the US Department of Energy Joint Genome Institute (JGI) and will include high‐quality drafts of 400 soybean accessions with reference‐quality assemblies of 50 of these lines. This project will provide four key benefits to soybean biology and agriculture: (1) It will allow immediate identification of agronomically important alleles, many of which are currently cryptic to GWAS approaches (e.g., structural variants that are not in LD with surrounding SNPs, such as the locus described by Fliege et al. [2022]). (2) It will uncover the structural and genetic diversity within the complex genomes of soybean and wild soybean relatives. (3) It will enable deep analyses of the evolution and domestication of modern soybean, through the application of haplotype‐based approaches and other methods requiring multiple whole‐genome assemblies. (4) It will empower soybean researchers and breeders to directly select for otherwise hidden genetic variation in genes that can be targeted for variety development. As soybean is becoming increasingly important as a worldwide crop, as well as being a key bioenergy crop, we hope this project will have a global impact and be particularly relevant to US agriculture. Expanding sequence information is key to understanding the human impact on the biology of soybean and its associated organisms, including pests, pathogens, and symbionts. Identifying resistance, susceptibility, virulence, and avirulence genes has immediate applications in breeding and can also accelerate type testing for many organisms and greatly enhance our understanding of evolution in the soybean ecosystem, giving early warning of potential breakdowns in crop protection.

The key anticipated outcome from this effort will be the establishment of an updatable pan‐genome data repository and tools, with the flexibility to incorporate new genomes as they become available.

## 8 COMPUTATIONAL RESOURCES

The soybean community has two highly active database teams working to develop tools and resources to support breeding, genetics, and genomics research. SoyBase, supported by the federal government and headquartered within the USDA‐ARS Corn Insects and Crop Genetics Research Unit in Ames, IA, has a stated goal to integrate genetics and genomics to advance soybean research. SoyKB, headquartered at the MU, serves as a web resource for soybean translational genomics. Given the vast amount of soybean genomics data being released on a daily basis, there is a critical need to support these public databases.

There are several other data services and websites that host genetic and genomic resources useful to the soybean community, including Phytozome, which serves as a plant comparative genomics portal run by the JGI. The JGI also houses data (https://genome.jgi.doe.gov/portal/) for several impactful pest species of soybean for the public. Furthermore, projects using soybean as a model to describe key developmental processes such as Gene Networks in Seed Development (http://seedgenenetwork.net/) are also replicated on SoyBase. For the sake of the discussion below, we have limited the scope of community accomplishments and upcoming priorities to the SoyBase and SoyKB platforms.

### 8.1 Computational resources—Recent accomplishments

#### 8.1.1 SoyBase recent accomplishments

SoyBase does not have a fixed release schedule, but new data are continually being added to the database and displays by SoyBase curators. Since the inception of the Soybean Genomics Research Program Strategic Plan in 2017, SoyBase has added seven soybean genomes and their associated gene models to the SoyBase Genome Browser (https://soybase.org/resources/). By making these data available, soybean researchers can visualize the sequence of the individual cultivars with known phenotypes and identify alleles associated with such phenotypes. Gene model representations in the Genome Browsers also display inferred molecular functional and process information. Additionally, the SoyBase team has added 40 *G. max* and six *G. soja* genomes and annotation sets for download on the SoyBase Data Store (https://soybase.org/data/v2/Glycine/max/ and https://soybase.org/data/v2/Glycine/soja/). These *Glycine* genomes and annotations originated from multiple publications (Chu et al., 2021; Garg et al., 2022; Liu et al., 2020; Xie et al., 2019; Zhuang et al., 2022). If available, variation data (SNPs) for each genome can also be downloaded. These diverse data will be used to help catalog soybean alleles, employing methods such as pan‐gene comparisons. Additionally, comparisons of wild perennial and annual soybean genomes to elite cultivars will help identify genomic regions associated with domestication. The SoyBase Gene Annotation Lookup Tool provides annotation information for user‐provided genes of interest. The SoyBase GO Term Enrichment Tool identifies overrepresented Gene Ontology (GO) terms in user‐provided lists relative to all predicted genes in the Wm82 genome.

Currently, SoyBase holds both pan‐genome assemblies and pan‐gene sets—the former representing genomic sequence across multiple accessions, and the latter representing genes across multiple accessions (https://soybase.org/tools/translate/). The pan‐genome described by Bayer et al. (2022) identifies sequences from the USDA core soybean germplasm that are either in common with or distinct from the Lee reference genome sequence. The pan‐genome described by Torkamaneh et al. (2021) identifies sequences from 204 diverse accessions of *G. max* that are not present in the reference assembly *G. max* ‘Wm82.a4’ (Valliyodan et al., 2019). The SoyBase group has generated a pan‐gene set for *Glycine*, currently comprising 56 annotation sets, based on sequenced *G. max* and *G. soja* accessions, and also showing correspondences with predicted genes from other *Glycine* species (https://data.legumeinfo.org/Glycine/GENUS/pangenes/). These data allow easy identification of corresponding genes between annotations, and they help to identify both widely shared and variant genes in the germplasm.

The Genome Comparison Visualization Tool (GCViT) can explore large genotyping datasets to identify conserved or divergent genomic regions and pedigrees (Wilkey et al., 2020). The Genome Context Viewer (GCV) is a tool to compare genome structure and will allow users to visualize genomic structural variation and gene CNV in multiple soybean genomes (Cleary & Farmer, 2018). In collaboration with the Plant Metabolic Network project (Hawkins et al., 2021), SoyBase has also updated the SoyCyc Soybean Metabolic Pathway Database. Using this database and Pathway Tools (Karp et al., 2020), users can visualize metabolic pathways and “paint” gene lists onto the pathways. This may provide biological insights into gene function.

To visualize gene expression patterns, the Gene Expression Explorer has access to over 60 Gene Expression Omnibus (GEO) experiments and provides graphical access to RNA expression experiments found in 10 different tissues and 19 different biotic and abiotic stresses. This assists soybean researchers in determining if candidate genes identified through QTL or GWAS analyses are expressed in tissues and developmental time points of interest, facilitating the identification of strong candidate genes. In collaboration with the University of Toronto (Waese et al., 2017), SoyBase displays gene expression in individual soybean structures and tissues based on publicly available gene expression experiments. This display can be useful in candidate gene validation and functional hypothesis generation. However, these represent only a small portion of the transcriptomic datasets available for soybean. As stated above, the magnitude of data released every month is proving a bottleneck for database integration.

SoyBase is collaborating with many groups to ensure that the data from the Uniform Soybean Tests (UST) are available for the community (https://www.soybase.org/uniform_trials.php). Phenotypic values for the Northern Uniform Soybean Tests (NUST) have been collected into a searchable database at SoyBase. Additionally, NUST and SUST (Southern Uniform Soybean Test) data will also be available from SoyBase, a subset of BreedBase, in the near future. SoyBase maintains a searchable database of soybean pedigrees including NUST and SUST entries from 1943 to the present and pedigrees of historical and elite cultivars. The SoyBase soybean pedigree database is used by university Intellectual Property departments and breeders in variety protection and patent applications.

SoyBase also serves as a communication and information center for soybean researchers. The SoyBase News section on the website homepage informs the community about updates to SoyBase, upcoming workshops, meetings, and conferences. Members of the community use this space to announce open position announcements. SoyBase also created and maintains the Soybean Breeders Workshop website.

#### 8.1.2 SoyKB recent accomplishments

In recent years, the SoyKB (https://soykb.org/) (Joshi et al., 2014, 2012, 2017) research team has focused on further optimization and improvements of its tools to support efficient analysis and utilization of large‐scale genomics and multiomics datasets generated by the community. SoyKB continues to provide a comprehensive web‐based framework for connecting soybean translational genomics, multiomics, and phenotypic datasets seamlessly to breeding. SoyKB framework updates are usually done on a monthly basis to add new multiomics datasets, and typically newly developed tools or feature enhancements for existing tools are implemented every year. The SoyKB framework offers a wide range of functionalities from basic data query, visualization, and download options, to performing computationally heavy tasks on analytic tools and pipelines to support the research community. These enhancements not only expedite the processing speed of existing functionalities but have also enabled the SoyKB team to make new tools available in the framework. Some of the tools developed and incorporated into SoyKB over recent years include AccuTool (Škrabišová et al., 2022), SNPViz 2.0 (Zeng et al., 2020; Zeng, Škrabišová, et al., 2021), Soybean Allele Catalog Tool (Chan, Dietz, et al., 2023), Genomic Variations Explorer (GenVarX) (Chan, Biová, et al., 2023), and Multiple Alleles discovery (MADis) (Biová et al., 2024) as described below. These tools are all powered by large‐scale genomic variation data from whole‐genome resequencing projects and phenomics data collected from various sources and processed by tools and pipelines, such as the variant calling pipeline (SnakyVC) (Chan, Dietz, et al., 2023) and Allele Catalog pipeline (AlleleCatalog) (Chan, Dietz, et al., 2023) developed by the SoyKB team.

The large‐scale soybean genomics data panels, Soy775 and Soy1066, that are used in AccuTool, SNPViz 2.0, Soybean Allele Catalog Tool, GenVarX, and MADis are from various sources including Zhou302v2 (Zhou et al., 2015), Liu304 (Liu et al., 2020), USB15x, USB40x (Valliyodan et al., 2021), Soja (Kim et al., 2010), and MSMC (Valliyodan et al., 2016).

AccuTool (Škrabišová et al., 2022) is a web‐based product of the implementation of the Synthetic Phenotype Association Study (SPAS) approach developed to enhance GWASs. Using AccuTool, researchers can detect genes in soybean that can formulate the Synthetic Phenotype to Causative Mutation (SP2CM) strategy. Through this methodology, more causative mutations of novel genes can be discovered effectively to evaluate the GWAS associations.

SNPViz v2.0 (Zeng et al., 2020; Zeng, Škrabišová, et al., 2021) is primarily focused on linking the GWAS results to genes and provides haplotype analyses. Using SNPViz 2.0, researchers can perform queries according to the chromosomal regions of interest for identifying haplotype blocks with SNPs and indels. In the visualization, hierarchical relations of accessions are presented in phylogeny trees outputted by phylogeny analyses. In addition, SNPViz 2.0 also supports additional statistical methods with *p*‐values, variant filtering, annotations, as well as color representations of SNPs and indels to assist researchers in expediting the causative genes discovery process that links back to their GWAS analysis.

The Soybean Allele Catalog Tool (Chan, Dietz, et al., 2023) is designed to enhance the visualization of the Allele Catalog datasets, connect causative alleles to phenotypes, and facilitate allele mining. Here, accession counts are grouped by unique allele combinations and corresponding accessions’ improvement status, as well as functional effects and amino acid changes of alleles in each chromosomal position. Results are clickable and linked to the phenotype viewer to visualize distributions of alleles and phenotypic traits for different alleles. Using this tool, researchers can gain more understanding of alleles to assist in selective breeding strategies for improvements in agricultural traits.

GenVarX (Chan, Biová, et al., 2023) is a toolset to investigate promoter regions and CNVs. The promoter region component is backed by TF binding sequence data, while the CNV component contains the CNV results from the Soy1066 data panel with cn.MOPs (Copy Number estimation by Mixture Of PoissonS) (Klambauer et al., 2012). Researchers can load a gene list of interest and visualize the promoter binding sites and gene binding sequences in the upstream regions of genes, sequence logos, and overlapping SNPs and indels in the gene binding sequences linked with phenotypes in the phenotype viewer. Researchers can also query genes or regions and view different copy numbers for each CNV region and view their phenotype distribution in the phenotype viewer. Using GenVarX, researchers can gain more understanding of genomic and structural variations and potential effects on soybean.

MADis is a tool that identifies multiple alleles and more importantly causative mutations (Biová et al., 2024). This tool uses extensive large‐scale GWAS datasets and computes a score for a combination of variant positions in a single candidate gene, and based on the highest score, it identifies the best number and combination of causative mutations. This tool shows how a genomic analysis can be employed to explore the natural and artificial selection of multiple alleles and, thus, improve and accelerate crop breeding in agriculture. This tool has also been developed and released as a Python package for users to perform this analysis on their own datasets, which will facilitate investigating combinatory variant positions on phenotypes.

### 8.2 Computational resources—Priorities (2024–2028)

SoyBase and SoyKB provide essential resources for the soybean community. Continued funding and support of these platforms is a shared top priority for the soybean research community. Upcoming priorities specific to the SoyBase and SoyKB teams are described below.

#### 8.2.1 SoyBase priorities

SoyBase was originally developed to house marker data. This continues to be an important resource for the soybean community. The team continually works to maintain and update the composite genetic map with biparental QTLs and the physical map with GWAS QTLs. To complement this resource, SoyBase is working to implement query and report systems to help identify allelic variants and marker–trait associations (including QTL ranges and GWAS marker associations). This tool will return a report of haplotypes (allelic variants) around a gene or specified region. Additionally, query and report systems will allow exploration of marker–trait associations and enable comparisons between marker–trait associations in different *Glycine* species and cultivars.

With the cost of sequencing still falling, SoyBase anticipates an avalanche of new genomic sequence data. SoyBase staff strongly encourage researchers to first submit data into one of International Nucleotide Sequence Database Collaboration (INSDC) databases when appropriate. For incorporation into SoyBase, datasets are first evaluated for their significance to the soybean research community. Data must then be acquired from the researchers or primary repositories and then processed for inclusion into SoyBase. Sequence data presented by SoyBase are available for download in its entirety without restriction. This will be true for any future data acquisitions.

SoyBase tools are currently available to allow selective sequence data retrieval from the data housed in the database. Some tools limit the amount of data that can be downloaded due to restrictions imposed by the HTTP protocol or by system constraints. In cases where extensive downloads are requested, they can be arranged by SoyBase staff if contacted by users.

In recognition that the datasets developed and utilized by the research community are continually evolving, SoyBase has begun to collect multiple omics datasets such as phenotypic, ionomic, metabolomic, and interactomic data. This requires SoyBase to effectively organize existing datasets of this raw data, stored elsewhere, for community access and utilization. The team is also working to expand the expression data available for researchers. scRNA experimental data are being integrated into the SoyBase infrastructure. These datasets will provide insight into the tissue and treatment expression patterns of the transcriptome and individual genes. SoyBase will review new tools and software being developed to visualize scRNA data, and until such a tool becomes available to meet SoyBase needs and environment, the Expression Explorer will be augmented with scRNA experiments. Visual display of these datatypes will be important, and SoyBase will evaluate software solutions for display that can be integrated into the local computing environment.

SoyBase will continue its efforts to curate gene models with functional and regulatory annotations. This effort will focus on Wm82 gene models and then can be extrapolated to other cultivars. Additionally, a set of legume‐focused gene families will be maintained and a phylogenetic tree viewer will be released to aid in identifying orthologous genes. This will complement efforts to maintain a set of genus‐level pan‐gene correspondences identified with “pandagma,” a program developed by the team. This will aid in identifying corresponding genes across genome sequences and annotations from different accessions. These “pan‐gene” sets can be identified through a combination of sequence similarity and synteny. Pan‐gene sets will facilitate an easy lookup of corresponding genes from different accessions and annotations, as well as a computed “composite” set of genes, derived from exemplar genes from all annotated accessions from the species.

The USDA has helped coordinate the annual UST (NUST and SUST) since the 1940s. The tests were coordinated by USDA personnel with cooperators from USDA and other public soybean programs. The results were published first as books and more recently as PDF files. These data record genetic improvements and the phenotypic advancement of soybean breeding in the United States and Canada. SoyBase has actively collected these data to directly support soybean breeders and breeding activities. In cooperation with the NCSRP SoyGen2 project, SoyBase has also collected genotypic profiles of all strains entered into the NUST since 1989. Phenotypes, coupled with the genotypic profiles, will be valuable for the development of AI methods to aid genomic selection strategies. Going forward, NUST and SUST cooperators will collect phenotypic data using standardized spreadsheets to facilitate incorporation into SoyBase. SoyBase and the Breeding Insight/BreedBase (BI/BreedBase) project have initiated a collaboration to capture the breeding and trial data from the NUST and enter it into a BI/BreedBase installation called SoyBase. BreedBase is a USDA‐supported project to serve as a breeding notebook and analysis platform, enabling a user to perform numerous statistical ad hoc calculations on the data, as well as genomic selection procedures. AI and genomic selection methods hold the promise to reduce the length of the breeding cycle and increase the rate of genetic gain in soybean. With detailed descriptions of the agronomic performance of strains, these data can serve as training sets necessary for AI algorithm development. This will speed the creation of superior cultivars to meet the increasing demand for plant‐based protein for human and animal consumption.

Furthermore, SoyBase curators will determine the parentage of each NUST/SUST line relative to PIs or until the parentage cannot be reasonably determined. This enables tracing the familial connections of not only the entries to the Uniform Tests but also historic registered cultivars. These data will also be useful to researchers who design GWASs, as close familial relationships can confound the results from those studies. It is also useful for university Intellectual Property departments to prepare agreements and register varieties with the Plant Variety Protection Office, and for the United States Patent Office to analyze patent submissions.

SoyBase will continue to provide ongoing support (website, email lists, elections, etc.) for the SoyGEC, Soybean Genetics Committee, Soybean Breeders Workshop, Biennial Soybean Meeting, and others as requested.

#### 8.2.2 SoyKB priorities

The SoyKB team has identified a need for tool development to address additional research areas and continue making further enhancements to existing tools for the future. The SoyKB team is currently working on further enhancing the MADis tool (Biová et al., 2024), which has been incorporated into SoyKB. This tool will compute the effects of genomic variations of multiple alleles using variant position combinations and statistical models, as the combined effects on phenotypes may not be necessarily caused by a single allele genomic variation in most cases but rather by the presence of parallel independent causative mutations. The MADis tool takes genes of interest and phenotype files from users to first compute the effects of two variant positions and rank variant position combinations that have high scores and explanatory percentages to compute the mutative effects of a combination of 1–7 variant positions.

The SoyKB team's priorities for the future include improving existing tools to extend the functionalities and capabilities and incorporation of newer datasets and data types. Expanding the existing Soy775 and Soy1066 soybean data panels and creating new data panels with the most current Wm82 reference genome (and other available reference genomes [Fiskeby III, Lee, *G. soja*]) are also planned. As more soybean whole‐genome re‐sequencing datasets become available, SoyKB plans to incorporate newer lines into the expanded curated panels and Allele Catalog tool, by doing updates one to two times a year. This will provide an opportunity for the community as an open call for submission of whole‐genome resequencing datasets of interest that they would like to have incorporated into this panel. Also, SoyKB plans to upgrade all tools to handle multi‐ and inter‐assembly searches considering the rapid development of more comprehensive genome assemblies, versions, and ultimately a soybean pan‐genome.

During the development of GenVarX (Chan, Biová, et al., 2023), the SoyKB team identified an urgent need for adding more complete information regarding annotated TFs in soybean, which is currently missing from one of the currently available public PlantTFdb databases. The number of TFs with complete binding information in this database is significantly underestimated and leaves a gap in information that is available for all annotated soybean TFs. SoyKB aims to create a more complete soybean‐specific TF database with binding information for motifs, which will feed complete details to the GenVarX tool. They also plan to build a protein‐level impact/effect tool to complement the Allele Catalog and Soy curated panels. Variants with predicted amino acid changes will be analyzed for protein sequence conservation using nonredundant sequences from other species and visualized with Weblogo (Crooks et al., 2004). A complementary aspect of the tool will be SIFT (Sim et al., 2012) scoring.

Additionally, the SoyKB team is also currently working on incorporating scRNA‐sequencing datasets within SoyKB and better integrating and connecting the SoyKB database and backend framework with the team's KBCommons framework to benefit from the newly developed 3D Omics Studio and “Cross‐Species and Comparative Multiomics Translation” (CCMT) tools in KBCommons. The CCMT tool provides a web‐based platform that offers comprehensive and interactive comparative functionalities between single and multiomics (transcriptomics, proteomics, metabolomics, miRNA, methylation, etc.) datasets for the same and between various organisms. This tool empowers researchers to compare differential expression across different species and different multiomics layers for organism‐specific analyses. The 3D Omics Studio tool provides researchers with an interactive and comprehensive platform for visualizing and exploring multiomics data, with a specific focus on differential expression analysis. Users can leverage this tool for seamlessly generating and visualizing UpSet and Venn plots, Kyoto Encyclopedia of Genes and Genomes (KEGG), GO, and Reactome enrichment analyses, Voronoi visualizations, KEGG Pathview Viewer, 2D Volcano plots, and PPI network representations on the fly.

The SoyKB team has also recently expanded G2PDeep‐v1 (Zeng, Mao, et al., 2021) to the newer G2Pdeep‐v2, which provides an all‐encompassing web‐based platform that harnesses the power of deep learning to offer comprehensive phenotype prediction and marker discovery analysis using six diverse types of multiomics data. Researchers can input one to three types of omics data in any combination into the application through an interactive interface to create and train deep learning models and predict phenotypes. It uses a fully automated hyperparameter tuning approach supported by high‐performance computing resources, and results can be accessed via an interactive visualization highlighting well‐trained model results for predicted phenotypes and markers. The SoyKB team plans to apply the G2PDeep‐v2 method to the expanded curated soybean data panels of Soy1066 and 622 bulk transcriptomics accessions study (Li et al., 2024) to perform phenotypic predictions and release the models with reliable performance and accuracy for public access to the soybean community, where they will be directly available for soybean researchers to utilize via SoyKB and KBCommons. The team also plans to further expand G2Pdeep‐v2 with the incorporation of scRNA‐sequencing datasets, so that such datasets from soybean can be used along with genomic variation details from various accessions for better phenotypic predictions.

Currently, the SoyKB framework provides limited support for end‐to‐end analysis from raw NGS sequencing data to analyzed results for direct incorporation into the database or tools, especially when the number of samples or the size of the data is large. Also, some of the tools may have restrictions on supporting certain genomic window ranges for searches, or on file sizes for data uploads or downloads, to ensure an optimized functioning and performance of the tools. The SoyKB team is addressing some of these limitations via a new KBCommons framework that supports all organisms and envisions an adoption of such enhanced capabilities into SoyKB in the future.

## 9 TRAINING THE NEXT GENERATION OF SOYBEAN RESEARCHERS

### 9.1 Realizing today's challenge for tomorrow's promise

Possibly the grandest opportunity to proactively shape the future of soybean research is to reach and engage young minds who will be the future in the field and who can provide solutions to evolving problems for farmers/growers, agriculture, and society at large. There are at least four stakeholders that stand to benefit from fostering this development. They include (i) students or early career scientists not yet engaged in soybean research, (ii) farmers/growers, (iii) scientists and enabling agencies at local, state, and national levels that have a vested interest, and (iv) agribusiness professionals including commercial entities directly tied to soybeans but also the manufacturers of renewable products. While farmers and those currently tied to soybean work are supportive of doing more, reaching tomorrow's potential scientists and enabling their curiosity through enhanced support by funding agencies will be crucial to blazing the trail of soybean research that is needed. Agricultural research, whether supported by federal agencies or commodity boards, can and must do better to engage minds in plant science and agricultural production that holds the key to future food wealth and a sustainable, renewable economy derived from plant products.

### 9.2 Invigorating current and future stakeholders

One of the current challenges is the shortage of young minds in the workforce to serve in scientific and industrial positions. We must find more avenues for engaging a new and diverse workforce, not just from traditional crop science fields but also from interdisciplinary fields including informatics, computer science, and AI to better address future challenges. A healthy amount of competition for good ideas and opportunities invigorates thought and maintains the highest quality of output. However, training requires a degree of continuity with students able to complete thesis projects prior to being hired by the industry. How can we establish a more thoughtful, forward‐thinking complementary relationship between the educational process and commercial agriculture entities that can provide the inspiration for a stable future career? Equally important is to widen the pool of trainees by ensuring that the soybean community is inclusive of historically marginalized groups.

### 9.3 Fellowships to inspire the minds of the next generation

One idea that evolved from the strategic planning meeting was to re‐envision fellowship programs for students. Imagine a fellowship program that incentivizes students with highly competitive salaries to recruit the best minds into soybean research. Support would come from commercial entities, commodity boards at state and national levels such as the United Soybean Board (USB) and state soybean boards, and hopefully also through potential partnerships with federal agencies such as the Foundation for Food and Agriculture Research (FFAR) to extend the reach of farmer‐based checkoff dollars. As one example, several companies could support a fellowship program that provides students with the chance to work consecutive summers while pursuing a Ph.D. at any of the commercial entities. The outcomes of such a program would include increased support for more students in soybean‐focused research areas deemed important to the above‐listed stakeholders. A program for postdoctoral research associates could be similarly envisioned. If the projects were reviewed and awarded by the commodity boards, then farmer interests would be paired with industry needs to equip the next‐generation workforce and scientific research community to meet new challenges.

Some of the proposed opportunities pair well with the interests of the current generation of students and therefore could compete favorably to recruit young people into science, particularly agriculture and biotech. The working group recognized the emerging use of AI in agriculture, deriving “climate‐smart” farming and agribusiness practices, as well as producing meaningful science to address climate change, including crop resilience and food security through synthetic biology for complex traits. What has made soybean a top crop in the United States remains true today: it is the highest protein‐producing seed and, in combination with significant oil production, represents a versatile crop, and as a legume, it requires fewer inputs and is more sustainable. This combination of unique traits, relative to other crops, positions soybean research as highly important among commodities. However, increases in soybean yield through breeding and biotechnology have strained protein levels, and evidence suggests that better leveraging of microbial symbionts will be necessary to continue to meet meal protein quality requirements established by the industry. Such efforts will also benefit the increasing growth in new markets within less well‐developed parts of the world that can help reduce food insecurity and fortify diets with protein and lipids.

## AUTHOR CONTRIBUTIONS

**Robert M. Stupar**: Conceptualization; funding acquisition; supervision; visualization; writing—original draft; writing—review and editing. **Anna M. Locke**: Conceptualization; writing—original draft; writing—review and editing. **Doug K. Allen**: Conceptualization; writing—original draft; writing—review and editing. **Minviluz G. Stacey**: Conceptualization; writing—original draft; writing—review and editing. **Jianxin Ma**: Conceptualization; writing—original draft; writing—review and editing. **Jackie Weiss**: Conceptualization; writing—original draft; writing—review and editing. **Rex T. Nelson**: Conceptualization; writing—original draft; writing—review and editing. **Matthew E. Hudson**: Conceptualization; writing—original draft; writing—review and editing. **Trupti Joshi**: Conceptualization; writing—original draft; writing—review and editing. **Zenglu Li**: Conceptualization; writing—original draft; writing—review and editing. **Qijian Song**: Conceptualization; writing—original draft; writing—review and editing. **Joseph R. Jedlicka**: Conceptualization; writing—original draft; writing—review and editing. **Gustavo C. MacIntosh**: Conceptualization; writing—original draft; writing—review and editing. **David Grant**: Conceptualization; writing—original draft; writing—review and editing. **Wayne A. Parrott**: Conceptualization; visualization; writing—original draft; writing—review and editing. **Tom E. Clemente**: Conceptualization; visualization; writing—original draft; writing—review and editing. **Gary Stacey**: Conceptualization; visualization; writing—original draft; writing—review and editing. **Yong‐qiang Charles An**: Conceptualization; visualization; writing—original draft; writing—review and editing. **Jose Aponte‐Rivera**: Conceptualization; writing—review and editing. **Madan K. Bhattacharyya**: Conceptualization; writing—review and editing. **Ivan Baxter**: Conceptualization; writing—review and editing. **Kristin D. Bilyeu**: Conceptualization; writing—review and editing. **Jacqueline D. Campbell**: Conceptualization; writing—review and editing. **Steven B. Cannon**: Conceptualization; writing—review and editing. **Steven J. Clough**: Conceptualization; writing—review and editing. **Shaun J. Curtin**: Conceptualization; writing—review and editing. **Brian W. Diers**: Conceptualization; writing—review and editing. **Anne E. Dorrance**: Conceptualization; writing—review and editing. **Jason D. Gillman**: Conceptualization; writing—review and editing. **George L. Graef**: Conceptualization; writing—review and editing. **C. Nathan Hancock**: Conceptualization; writing—review and editing. **Karen A. Hudson**: Conceptualization; writing—review and editing. **David L. Hyten**: Conceptualization; writing—review and editing. **Aardra Kachroo**: Conceptualization; writing—review and editing. **Jenny Koebernick**: Conceptualization; writing—review and editing. **Marc Libault**: Conceptualization; writing—review and editing. **Aaron J. Lorenz**: Conceptualization; writing—review and editing. **Adam L. Mahan**: Conceptualization; writing—review and editing. **Jon M. Massman**: Conceptualization; writing—review and editing. **Michaela McGinn**: Conceptualization; writing—review and editing. **Khalid Meksem**: Conceptualization; writing—review and editing. **Jack K. Okamuro**: Conceptualization; writing—review and editing. **Kerry F. Pedley**: Conceptualization; writing—review and editing. **Katy Martin Rainey**: Conceptualization; writing—review and editing. **Andrew M. Scaboo**: Conceptualization; writing—review and editing. **Jeremy Schmutz**: Conceptualization; writing—review and editing. **Bao‐Hua Song**: Conceptualization; writing—review and editing. **Adam D. Steinbrenner**: Conceptualization; writing—review and editing. **Benjamin B. Stewart‐Brown**: Conceptualization; writing—review and editing. **Katalin Toth**: Conceptualization; writing—review and editing. **Dechun Wang**: Conceptualization; writing—review and editing. **Lisa Weaver**: Conceptualization; writing—review and editing. **Bo Zhang**: Conceptualization; writing—review and editing. **Michelle A. Graham**: Conceptualization; funding acquisition; supervision; writing—original draft; writing—review and editing. **Jamie A. O'Rourke**: Conceptualization; funding acquisition; supervision; writing—original draft; writing—review and editing.

## CONFLICT OF INTEREST STATEMENT

The authors declare no conflicts of interest.

## Data Availability

Data summarized and associated with this article are provided in the figures and tables. Referenced data are available in the literature.
